# Early maturation of host antimicrobial peptide defences is associated with host–pathogen coexistence

**DOI:** 10.1038/s41589-026-02254-6

**Published:** 2026-07-15

**Authors:** Phillip A. Jervis, Gonçalo M. Rosa, Kieran A. Bates, Kersti Karu, Aamina Murtza, Thomas Defuentes, David Firer, Sam Edwards, Kevin Hopkins, François Le Mauff, Christopher J. Michaels, Tresai Jordine-Morgan, Christina Nylander, Christopher Sergeant, Emily J. Skelly, Tam T. Bui, Donald Sheppard, Xavier A. Harrison, Johanna Rhodes, Benjamin Tapley, Jaime Bosch, Trenton W. J. Garner, Matthew C. Fisher, Alethea B. Tabor

**Affiliations:** 1 Institute of Zoology, Zoological Society of London, London, UK; 2 MRC Centre for Global Infectious Disease Analysis, School of Public Health, Imperial College, London, UK; 3 Department of Chemistry, UCL, London, UK; 4 IMIB Biodiversity Research Institute (CSIC-Oviedo University-Principality of Asturias), Mieres, Spain; 5 Centre for Ecology, Evolution and Environmental Changes (cE3c) and Global Change and Sustainability Institute (CHANGE), Faculdade de Ciências da Universidade de Lisboa, Lisbon, Portugal; 6 Blizard Institute, The Faculty of Medicine and Dentistry, Queen Mary University of London, London, UK; 7 Department of Microbiology and Immunology, McGill University, Montreal, Québec Canada; 8 Infectious Diseases and Immunity in Global Heath Program, Research Institute of the McGill University Health Centre, Montreal, Québec Canada; 9 Department of Plant and Environmental Sciences, University of Copenhagen, Frederiksberg, Denmark; 10 Living Systems Institute, University of Exeter, Exeter, UK; 11 Laboratory of Virology, Wageningen University and Research, Wageningen, The Netherlands; 12 Zoological Society of London, London, UK; 13 Centre for Biomolecular Spectroscopy and Randall Division of Cell and Molecular Biophysics, King’s College London, New Hunt’s House, London, UK; 14 Centre for Ecology and Conservation, University of Exeter, Penryn, UK; 15 Radboud University Medical Centre, Department of Medical Microbiology, Nijmegen, The Netherlands; 16 School of Biosciences, University of Birmingham, Birmingham, UK; 17 Unit for Environmental Sciences and Management, Northwest University, Private Bag X6001, Potchefstroom, South Africa

**Keywords:** Chemical ecology, Mass spectrometry, Infectious diseases, Proteomics

## Abstract

Epizootic outbreaks of invasive pathogens, such as the fungal pathogen *Batrachochytrium* *dendrobatidis* (*Bd*), are increasingly recognized as a global threat to biodiversity. However, not all susceptible host populations are equally impacted by these disease outbreaks. While some populations persist despite intense infections and high mortality rates, others can transition to enzootic coexistence with the pathogen. Here, we use comparative peptidomics to identify differences in antimicrobial peptide (AMP) maturation in *Bd*-susceptible common midwife toads (*Alytes* *obstetricans*) through metamorphosis. Here, we show that animals that metamorphose before AMP immune maturation display a low-diversity AMP phenotype deficient in anti-*Bd* AMPs. Where a high incidence of postmetamorphic animals occur with immature AMPs, populations are associated with epizootic disease dynamics. Conversely, populations associated with mature AMPs are stable following invasion of the pathogen. Our results show that even intrinsically susceptible amphibian species can possess the tools that allow populations to recover following a severe *Bd* epizootic.

**Figure:**
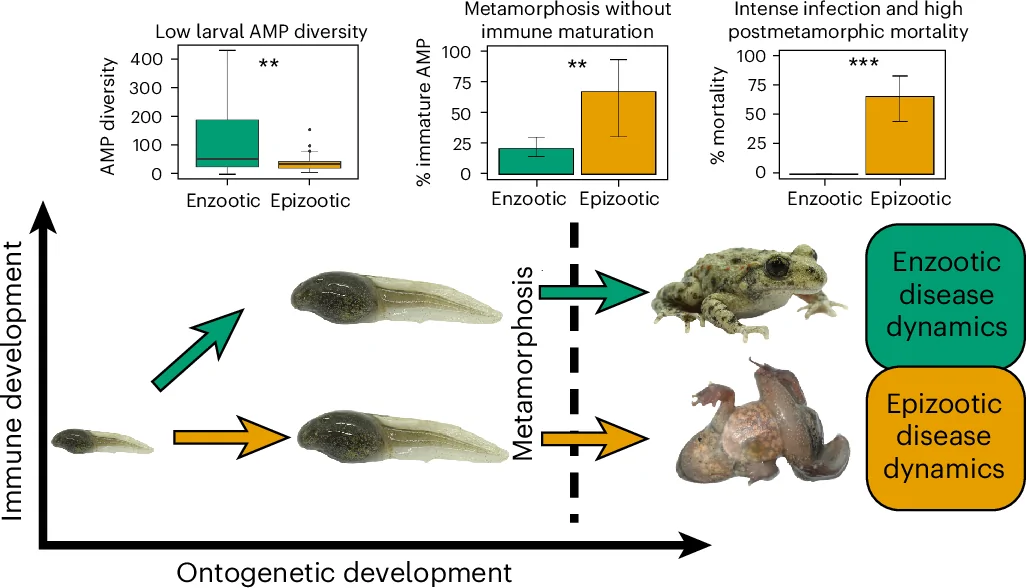

## Abstract (web summary)

Host antimicrobial peptides (AMPs) are a frontline defense against infection. Now, it has been shown that maturation of AMP repertoires early in life can predict stable host coexistence with a highly invasive pathogen.

## Main

When highly transmissible pathogens are introduced into a naive community of susceptible hosts, these often experience one or a series of epizootic waves^1^. Over time, barring extinction of the pathogen or the host, epizootic disease dynamics will usually subside into more stable enzootic equilibria. Divergent trajectories and asynchronous rates of recovery can generate contemporary patterns of epizootic and enzootic disease dynamics between persisting host populations^2,3^. Comparative studies between these populations can offer insights into the role of host immunity in shaping population-level disease outcomes^4^. Antimicrobial peptides (AMPs) are a crucial and near-ubiquitous component of innate immune defense across the tree of life^5^ and have been shown to inhibit the growth of bacterial, viral and fungal taxa in vitro^6^. Innate immune effectors, including AMPs, are traditionally considered a primitive, broad-spectrum antimicrobial barrier for preventing infection^7^. Despite this long-held perception, there is a growing body of work challenging this oversimplification^5,8^. While the innate immune system does constitute the first line of defense against novel pathogens, the complexity and specificity of the constituent AMPs has been long overlooked.

As anthropogenically spread emerging infectious diseases (EIDs) increasingly impact humans, agriculture and wildlife^9^, there is a pressing need to understand the interactions between host immunity and these novel pathogens. Here, the amphibian-infecting chytrid fungus, *Batrachochytrium* *dendrobatidis* (*Bd*), is one of the most impactful EIDs known and is a recognized threat to amphibian diversity worldwide^10^. Chytridiomycosis, the disease caused by *Bd*, causes acute epidermal disruption, leading to pathophysiological changes associated with high rates of mortality in susceptible species^11^. The resulting population declines have been monitored over the past forty years and the loss of amphibian taxa, particularly in tropical montane ecosystems, results in the loss of ecosystem function^12,13^ and the collapse of predator communities through trophic cascades^14^. Despite these localized catastrophic events, at a global level, the emergence of *Bd* is associated with heterogeneous patterns of declines of susceptible amphibians at both interspecific^11^ and intraspecific levels^2,3^.

There is some evidence to suggest that increased host immunity is contributing to the persistence of susceptible host populations^11,15^. As a pathogen of amphibian skin, *Bd* must first survive contact with the epidermal mucosal layer before it can infect the host. Variation in the efficacy of these mucosal defenses may explain the observed heterogeneity in population-level responses to a *Bd* epizootic. The highly diverse mixtures of both host^16^ and microbial compounds^17^ present in the mucosal layer have been shown to inhibit the growth of *Bd* in vitro^18^ and in vivo^15^. AMPs, in particular, are thought to be a critical component of an amphibian’s mucosal innate immune defense against pathogens such as *Bd*. This hypothesis owes much to previous in vitro work showing increased anti-*Bd* efficacy of mucosal samples from resistant amphibian species and the corresponding in vitro activity of synthetic amphibian AMPs^19^. However, there has been substantial difficulty in linking these in vitro AMP experiments to population-level studies in the field.

In many amphibian species, the metamorphosis from the larval to postmetamorphic stages carries an elevated risk of mortality from chytridiomycosis^20^. This elevated risk is generated through two simultaneous developmental processes, keratinization of the skin and immune maturation. As a keratinophilic parasite, *Bd* requires keratinized amphibian skin as a substrate for its survival in vivo^21^. In fatal chytridiomycosis, the osmoregulatory capacity of the amphibian’s skin breaks down, leading to cardiac asystolic death through electrolyte imbalance^11^. However, tadpoles do not possess keratinized skin^22^. Whilst *Bd* can infect their keratinized mouthparts, this does not develop into chytridiomycosis and is generally not fatal^23^. During metamorphosis, as keratinization of the skin occurs, the pathogen can spread to the skin, which can then develop into chytridiomycosis. This transition from a *Bd*-tolerant to a *Bd*-susceptible life stage coincides with a critical period of immune reorganization^24^. The immune system of tadpoles is considered immature and less effective in comparison to postmetamorphic animals^25^. At an organismal level, the development of the constitutive, innate and adaptive immune defenses takes places at discrete stages before, during and after metamorphosis as the requisite organs develop^26^. At a skin level, the AMP repertoire of tadpoles is considerably different to postmetamorphic life stages^25,27^, with further changes potentially occurring between recent metamorphs and sexually mature adults^28^. The inhibitory potential of these limited AMP defenses in larval amphibians has been reported to be significantly lower than those of postmetamorphic animals^27^. However, there has been no research into the population-level variation of these immune profiles across life stages in nature.

The common midwife toad (*Alytes* *obstetricans*) is susceptible to *Bd* but its population-level response to *Bd* introduction has been highly variable^2,29–31^. This species is particularly susceptible to chytridiomycosis during or shortly after metamorphosis, often leading to seasonal mass mortality events in montane regions^31^. In the Western Pyrenean Mountain range, all populations of *A*. *obstetricans* experienced heavy declines following *Bd* introduction, yet four of these populations have persisted 19 years later, with three transitioning to stable, enzootic disease dynamics^2^. At Lac d’Arlet, one population continues to exhibit epizootic dynamics characterized by high *Bd* infection intensity, seasonal mass mortality events and near extirpation. This observed heterogeneity in disease dynamics is known to be associated with amongst-lake variation in host microbiome^2,4^; however, an underlying immunological mechanism driving these host–microbe interactions is missing.

Here, we aimed to investigate the extent to which the observed disease dynamics were associated with variation in host AMP defenses. The AMP defenses of *A*. *obstetricans* include the alyteserin family, which consists of 12 cationic, α-helical cytolytic AMPs that are C-terminally amidated^32^. However, it remains unknown whether some or all the known alyteserins inhibit *Bd* or whether they could operate synergistically as is the case in other amphibian AMP systems^33,34^. If there is variation in anti-*Bd* efficacy between AMPs, then population-level responses to chytridiomycosis could occur through differences in composition or concentration of their AMP arsenal.

Since the description of the alyteserin AMPs in *A*. *obstetricans* in 2009, recent advances in mass spectrometry (MS)-based proteomics^35,36^, when integrated with transcriptomic references, has allowed for the high-throughput characterization of complex peptidomic mixtures. While these methods are typically used in human health research^37,38^, we were able to apply these analytical techniques to this wild amphibian disease system. In combination with molecular diagnostics, in vitro AMP *Bd* inhibition assays and confocal microscopy, we leveraged a multidisciplinary approach to investigate whether these differences in contemporary disease dynamics in the Western Pyrenees are associated with (1) the composition and efficacy of host AMP defenses in recently metamorphosed animals; (2) preinvestment in AMPs at the larval stage; and (3) the varied epidemiological outcomes that we observe across this long-term study system.

## Results

**Fig. 1:**
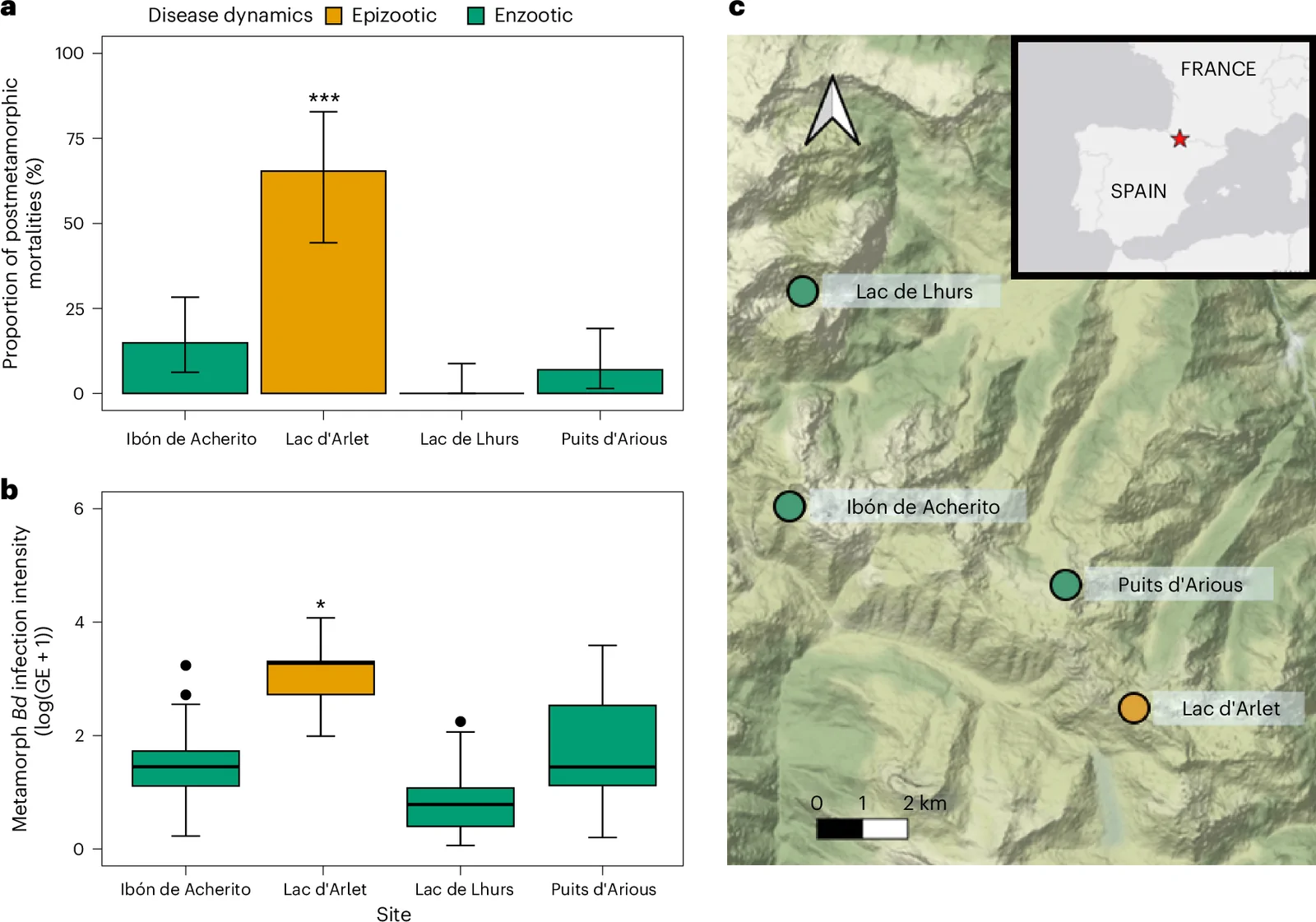
Post metamorphic *Bd* disease dynamics in the Western Pyrenees. **a**, Proportion of deceased postmetamorphic individuals (Ibón de Acherito, *n* = 7; Lac d’Arlet, *n* = 17; Lac d’Lhurs, *n* = 0; Puits d’Arious, *n* = 3) from total encountered (Ibón de Acherito, *N* = 47; Lac d’Arlet, *N* = 26; Lac d’Lhurs, *N* = 40; Puits d’Arious, *N* = 43) during field surveys. Significance was determined by Kruskal–Wallis test (*χ*^2^(3) = 58.497, *P* < 0.001), followed by a pairwise, two-sided Fisher’s exact test with Hochberg correction for multiple comparisons (*P* < 0.001). Error bars are Clopper–Pearson confidence intervals at 95% confidence. **b**, Box plot showing that the infection intensity of *Bd*-positive animals (*n* = 5) at Lac d’Arlet (Epizootic) is significantly higher than infection burdens in the enzootic populations (Ibón de Acherito, *n* = 33; Lac d’Lhurs, *n* = 30; Puits d’Arious, *n* = 38). Significance was determined using a Kruskal–Wallis test, followed by a pairwise, two-sided Wilcoxon test. **c**, Map of field sites. All individuals represent biological replicates. The box plot shows the median (center), 25th and 75th percentiles (box limits) and 1.5× the interquartile range (whiskers). Significance is denoted by *P < 0.05, **P < 0.01 and ***P < 0.001. The map image is the intellectual property of Esri and its licensors and is used herein under license (Copyright © 2021 Esri and its licensors). Source data

We confirm the continuation of epizootic disease dynamics, characterized by high levels of mortality (Fig. 1a) and infection intensity (Fig. 1b), in recently metamorphosed *A*. *obstetricans* at Lac d’Arlet. While this population has continued to persist despite many years of heavy chytridiomycosis-driven mortality, the low numbers of postmetamorphic individuals found during surveys (2019: *n* = 2, 2020: *n* = 7) relative to the enzootic populations (Ibón de Acherito, Puits d’Arious and Lac de Lhurs) shows that the population has yet to successfully coexist with the introduced pathogen.

### The mucosal peptidome is a highly diverse immunophenotype

**Fig. 2:**
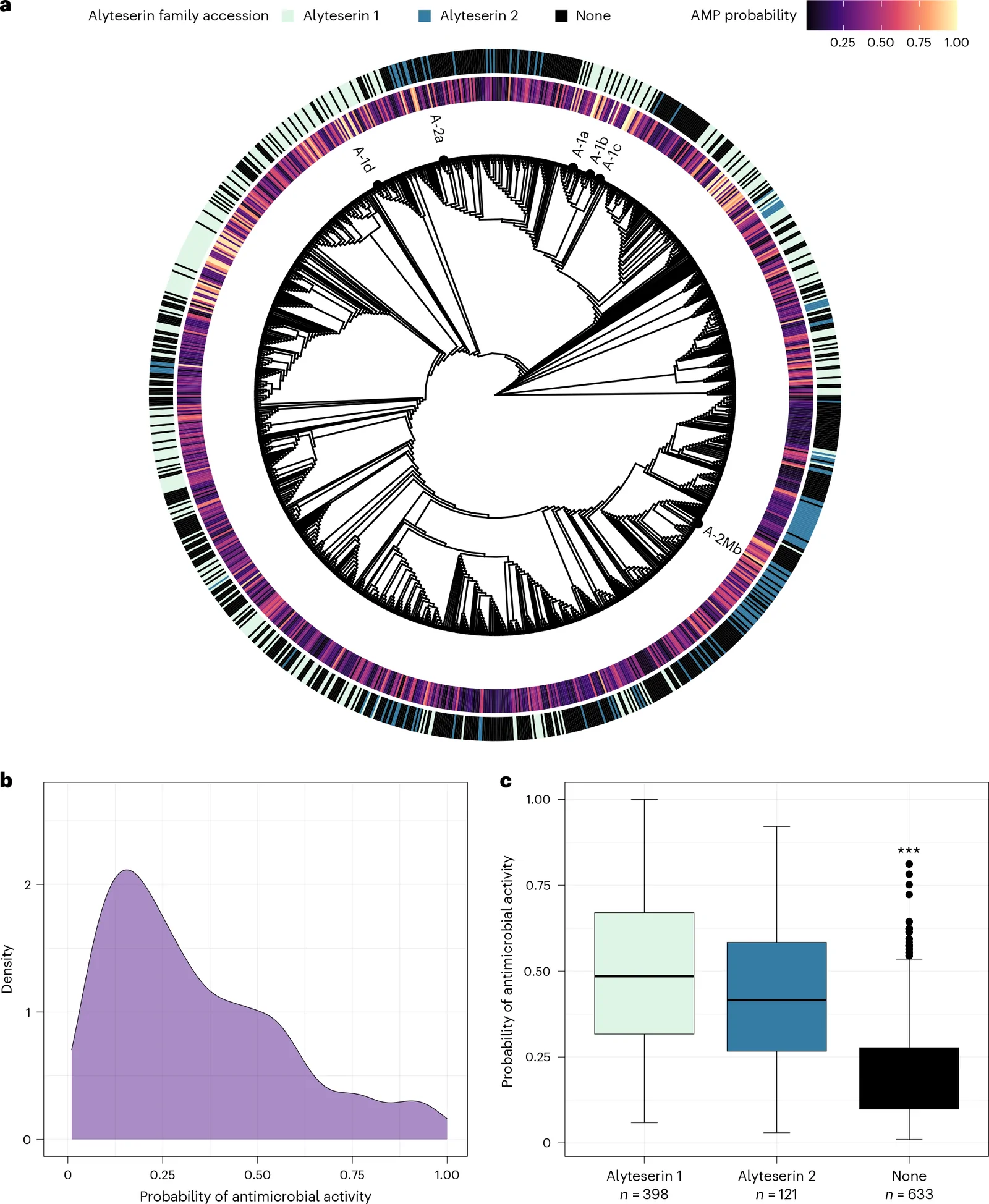
Peptidomic characterisation of skin-secreted peptides in *A. obstetricans*. **a**, Cladogram showing the 1,152 mucosal peptides characterized from the field data. Known AMPs are annotated. The inner heat map indicates the probability that each sequence has antimicrobial activity (as predicted by MACREL AMP prediction tool). The outer heat map shows alignment of each peptide to alyteserin AMP family. **b**, Frequency distribution of the probability of AMP activity predicted by MACREL. **c**, Box plot showing significantly lower predicted antimicrobial activity of peptides not aligned to alyteserin-family AMPs. Significance was determined using a Kruskal–Wallis test (*χ*^2^(2) = 448.83, *P* < 0.001), followed by a pairwise, two-sided Wilcoxon test with Benjamini–Hochberg correction for multiple comparisons (*P* < 0.001). The box plot shows the median (center), 25th and 75th percentiles (box limits) and 1.5× the interquartile range (whiskers). Significance is denoted by *P < 0.05, **P < 0.01 and ***P < 0.001. Source data

We sampled skin secretions from a total of 154 *A*. *obstetricans* tadpoles and 123 postmetamorphic individuals across the four survey sites. Peptidomic analysis of the samples was then carried out by liquid chromatography (LC)–MS/MS under conditions that allowed for detection and sequencing of all peptide components. To identify these peptides, we performed a peptidomic workflow in PEAKSStudio12 (Bioinformatic Solutions). In summary, de novo peptide sequences went through a two-round database search (first against the AMP database (APD3 database^39^) and then against a skin transcriptome reference library generated from captive *A*. *obstetricans*). All unaligned sequences were then assembled into de novo features using the DeepNovo algorithm^40^, before quality filtering and quantification of all identified peptide features. We found that the mucosal peptidome of *A*. *obstetricans* skin is far more diverse than the existing literature suggests (Fig. 2a)^32^. The field mucosal peptide samples yielded 1,820 putative peptides (inclusive of post-translational modifications), with 1,152 of these peptides passing the quality filtering stage for label-free quantification (Fig. 2a). Amongst these peptides are six previously described alyteserin AMPs (Supplementary Table 1)^32^. We also found alyteserin 2 Mb, which was only previously described in the closely related *A*. *maurus*^41^. Overall, from the 1,152 secreted mucosal peptides, the majority of these peptides were not predicted to have antimicrobial activity (Fig. 2b) by the MACREL AMP prediction tool^42^ (mean probability of AMP activity: 0.34, range: 0.01–1, s.d.: 0.24).

To enrich the dataset for peptides likely to possess antimicrobial activity, we removed peptide sequences without significant (−log*P* > 20) alignment to alyteserin 1 or alyteserin 2 AMPs (Fig. 2c). These removed sequences were predicted to have a significantly lower probability of AMP activity (Kruskal–Wallis: *χ*^2^(2) = 448.83, *P* < 0.001; pairwise Wilcoxon test: *P* < 0.001). A surprisingly large number of peptides had alignment with alyteserin 1 (*n* = 398) or alyteserin 2 (*n* = 121) AMPs, which is far higher than expected given the limited diversity of reported AMPs in the species. Much of this diversity appears to have been introduced after translation. This variation includes C-terminal acid and C-terminal amide analogs of all alyteserin AMPs, fragments of alyteserin AMPs and likely variation in the cleavage site from the propeptide observed as additional amino acids at either the C or N terminus of the AMP. This post-translational processing generates a large diversity of alyteserin peptide variants (alyteserin-PVs).

### Alyteserin AMPs show variable ability to inhibit *Bd* in vitro

To investigate whether the AMPs from *A*. *obstetricans* represent an effective defense against *Bd*, we performed in vitro *Bd* growth inhibition assays against a panel of known AMPs in the species. This included all seven previously described alyteserin 1 and alyteserin 2 AMPs described previously^32^ (two of which were not found in our study system) and alyteserin 2 Mb (which we found in *A*. *obstetricans* within this study). Despite their reported universal efficacy against bacteria^32,41^, they showed variable ability to inhibit the growth of *Bd* in vitro (Supplementary Table 2). Only alyteserin 1a (half-maximal effective concentration (EC_50_) = 8.47 μmol), alyteserin 1b (EC_50_ = 41.03 μmol), alyteserin 2c (EC_50_ = 37.14 μmol) and alyteserin 2 Mb (EC_50_ = 7.53 μmol) were found to be inhibitory against *Bd*.

**Fig. 3:**
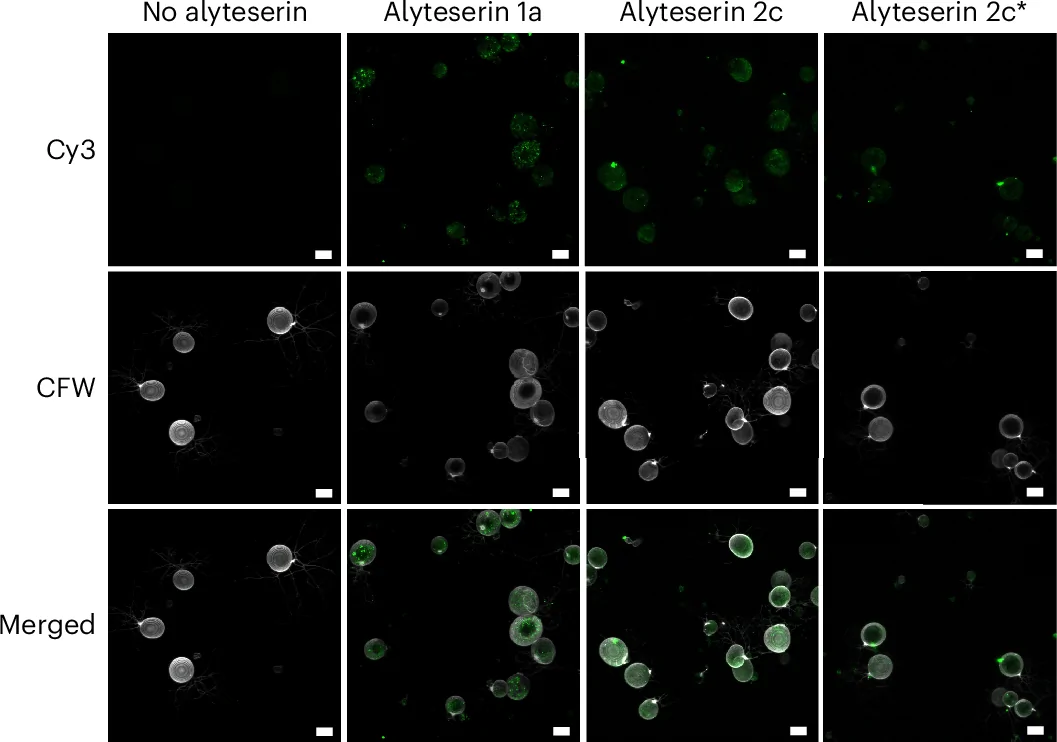
Confocal microscopy experiment of *Bd*-GPL isolate IA042 challenged with Cy3-labeled peptides. The four treatments were negative control, Cy3–alyteserin 1a, Cy3–alyteserin 2c and Cy3–alyteserin 2c (scrambled sequence). Imaging channels shown are Cy3 (alyteserin AMP, green) and Calcofluor white (cell walls, white) and the merged channel of both dyes. Imaging shows that alyteserins interact with *Bd* through an intracellular mechanism. The asterisk indicates scrambled peptide sequence. Scale bar, 10 µm. Each experiment was repeated twice and the same observations were made in both experiments.

We then carried out a preliminary investigation into elucidating the mode of action of alyteserin AMPs against *Bd*. It is generally thought that AMPs from frog skin secretions exert their antibacterial activity by disruption of the bacterial membrane^43^. Both the alyteserins and the temporins contain amphipathic sequences that adopt α-helical structures in membranes and in membrane-mimicking environments, leading to cell lysis; furthermore, changes in the sequences that promote helicity and membrane interactions enhance antibacterial activity^44^. The activity of temporin family peptides against *Bd* has also been hypothesized to depend on membrane disruption. An all-D-analog of temporin A was found to adopt an α-helical conformation in trifluorethanol (TFE) and had enhanced potency against *Bd*^16^,suggesting a membrane disruption rather than a receptor-binding mode of action. As similar structure–activity studies were not previously carried out with the alyteserins, we synthesized two of the most potent analogs (alyteserin 1a and alyteserin 2c) and a scrambled analog (scr-alyteserin 2c) as a control to study their conformational properties (Supplementary Documents 1 and 2). Circular dichroism (CD) measurements in both TFE and in 10 mM SDS showed that alyteserin 1a adopted a predominantly α-helical conformation in both solvents. This was less pronounced for alyteserin 2c and scr-alyteserin 2c had little or no secondary structure (Supplementary Document 3). To further probe the binding localization of the alyteserin informing on potential mode of action, we stained killed *Bd*-GPL isolate IA042 against Cy3-labeled versions of Alyteserin 1a, alyteserin 2c and scr-alyteserin 2c (Supplementary Document 1). Contrary to expectations, both alyteserin 1a and alyteserin 2c appeared to stain intracellular structures of *Bd* inside both the mature zoosporangia and the encysting zoospores (Fig. 3). The control scrambled alyteserin 2c did not expose such staining patterns, as only random fluorescent puncta were observed across the imaging areas. As these puncta did not seem to consistently locate with a killed chytrids, such signals were considered as background signal (Fig. 3). Collectively, the exclusive intracellular localization of the active versions of alyteserin suggests an intracellular inhibition mode of action involving either a specific partner or an interaction with intracellular membranes (Fig. 3).

### Reduced peptide diversity is associated with epizootic disease dynamics

**Fig. 4:**
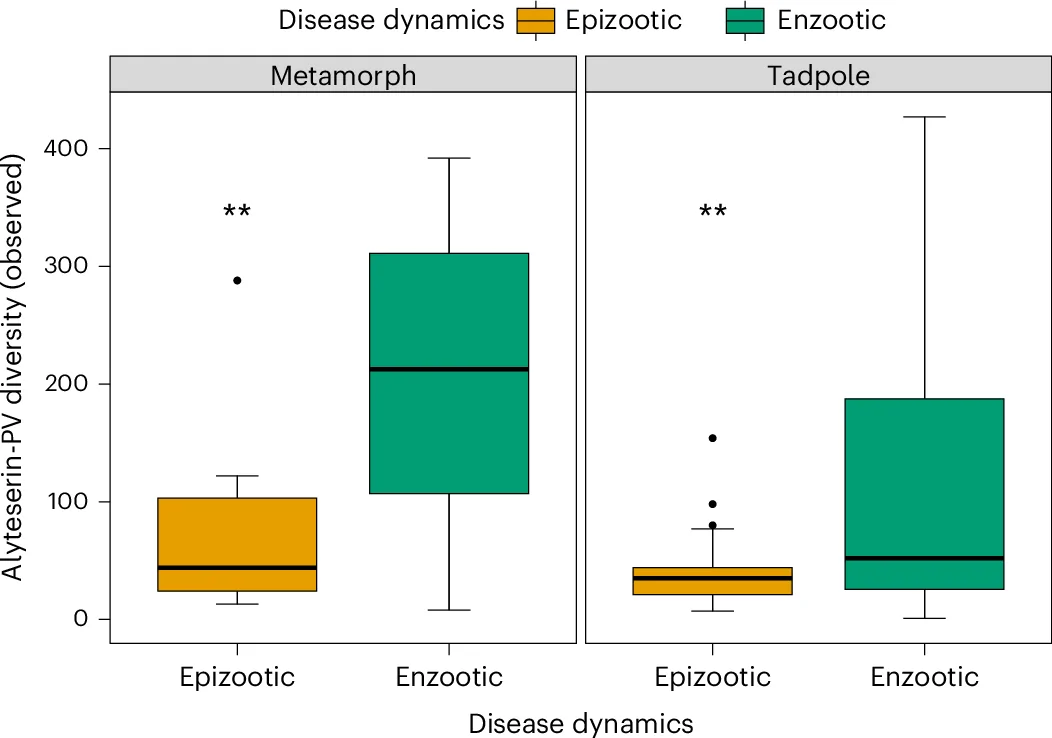
Epizootic disease dynamics are associated with lower alyteserin-PV diversity. Box plot showing that *A*. *obstetricans* at the epizootic Lac d’Arlet population has a significantly lower alyteserin-PV diversity at both postmetamorphic (Ibón de Acherito, *n* = 40; Lac d’Arlet, *n* = 9; Lac d’Lhurs, *n* = 37; Puits d’Arious, *n* = 37) and larval (Ibón de Acherito, *n* = 37; Lac d’Arlet, *n* = 43; Lac d’Lhurs, *n* = 40; Puits d’Arious, *n* = 34) life stages than in the enzootic populations. Box plots show the median (center), 25th and 75th percentiles (box limits) and 1.5× the interquartile range (whiskers). All individuals represent biological replicates. Significance was determined using a two-sided Wilcoxon rank-sum test for larval (*W* = 3,095, *P* = 0.004) and postmetamorphic (*W* = 208, *P* = 0.003) life stages. Box plots show the median (center), 25th and 75th percentiles (box limits) and 1.5× the interquartile range (whiskers). Significance is denoted by *P < 0.05, **P < 0.01 and ***P < 0.001. Source data

We found that the alpha diversity of alyteserin-PVs was significantly lower in the epizootic population compared to the three enzootic populations (Fig. 4) across both larval (Wilcoxon test: *W* = 3095, *P* = 0.004) and postmetamorphic (Wilcoxon test: *W* = 208, *P* = 0.003) life stages. We then investigated whether these trends in alyteserin-PV diversity were associated with developmental differences in the restructuring of the skin-secreted peptidome through metamorphosis.

### Maturity of AMP defenses is not intrinsically linked to metamorphosis

**Fig. 5:**
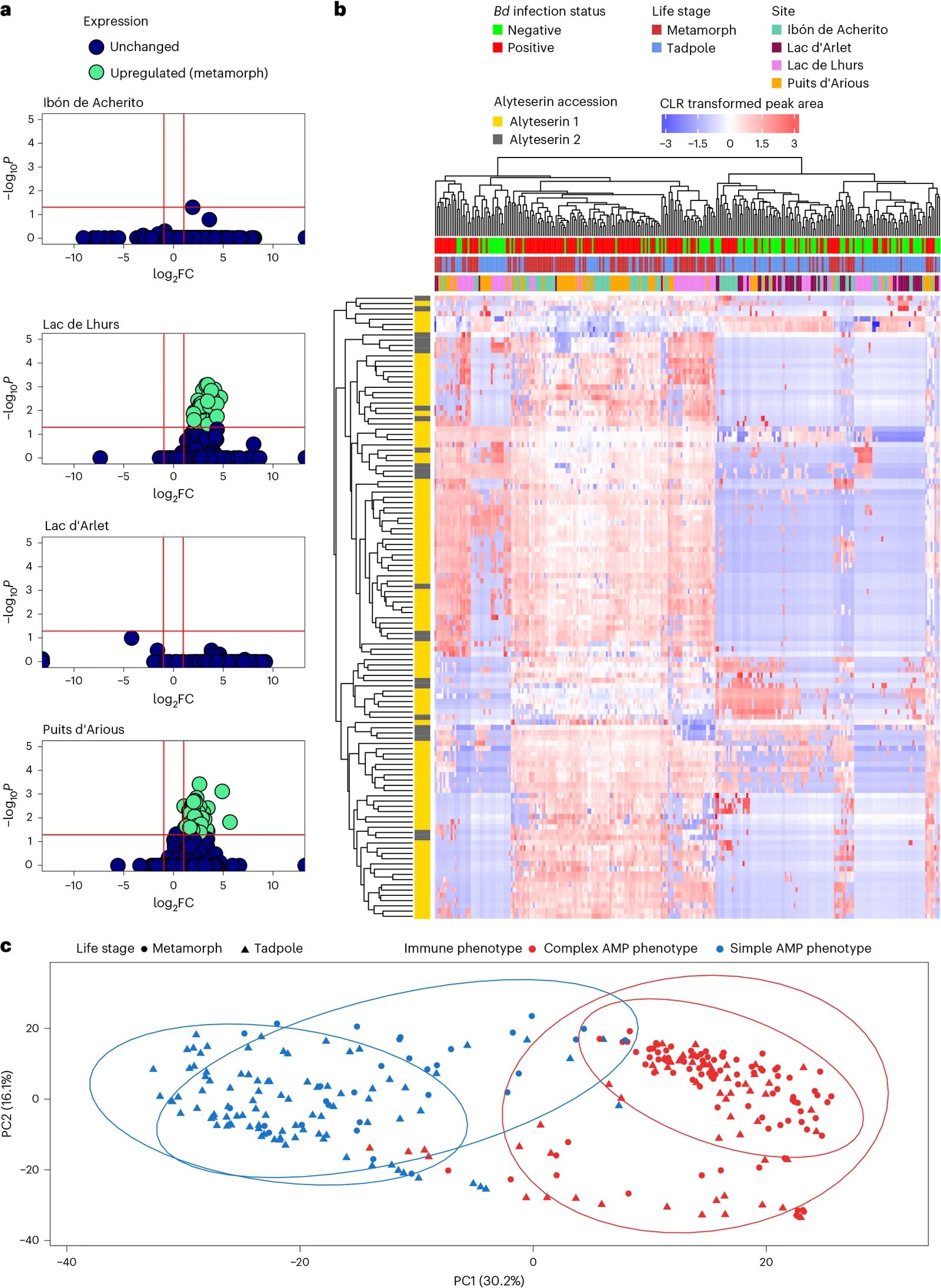
AMP maturation across four populations of *A. obstetricans*. **a**, Differential abundance analysis of Alyteserin-aligned peptides between life stages in each population. Significance was determined using a Wilcoxon rank-sum test at *P* < 0.05, with Hochberg correction for multiple comparisons. FC, fold change. **b**, Heat map showing that the clustering of differentially abundant peptides does not partition by life stage. Instead, there is a cluster with a high relative abundance of AMPs (mature AMP phenotype; left) and a cluster with a low relative abundance of AMPs (immature AMP phenotype; right). **c**, PCA showing that cluster membership, not life stage, is the strongest predictor of secreted peptide composition. All individuals represent biological replicates. Source data

To characterize the changes in the skin-secreted AMP defenses through metamorphosis, we performed differential abundance analysis of the alyteserin-PVs between larval and postmetamorphic life stages within each population. We found that the numbers of differentially abundant peptides (log_2_ fold change > 1, Hochberg corrected *P* value < 0.05) varied substantially between populations (Fig. 5a). There were a total of 121 unique alyteserin-PVs with significantly increased abundance in postmetamorphic individuals at Puits d’Arious (*n* = 98) and Lac de Lhurs (*n* = 58). However, there were no differentially abundant peptides between life stages at either Lac d’Arlet or Ibon de Acherito. We then performed a hierarchical, complete-linkage clustering analysis on these differentially abundance peptides (Fig. 5b). Surprisingly, the two clusters identified by the basal node of this analysis did not clearly separate larval and postmetamorphic life stages. Instead, each of these two clusters comprised a mixture of both life stages. The first cluster comprised mostly the postmetamorphic life stage (123/184 individuals) and was characterized by a higher compositional abundance of the differentially abundant secreted peptides. The second cluster comprised mostly the larval life stage (93/123 individuals) and was characterized by a lower compositional abundance of the differentially abundant secreted peptides. We assigned these clusters as ‘complex AMP phenotype’ and ‘simple AMP phenotype’, respectively.

We then performed a principal component analysis (PCA) to investigate the relative contributions of life stage, cluster membership and *Bd* infection status on the composition of these differentially abundant peptides (Fig. 5c). We found that cluster membership was a substantially stronger predictor of secreted peptidome composition than life stage or *Bd* infection (PERMANOVA: cluster membership, *R*^2^ = 0.24, *P* < 0.001; life stage, *R*^2^ = 0.02, *P* < 0.001; *Bd* infection status, *R*^2^ = 0.01, *P* < 0.01; cluster membership × life stage, *R*^2^ = 0.01, *P* < 0.001).

### Immature AMP phenotypes are deficient in anti-*Bd* AMPs

**Fig. 6:**
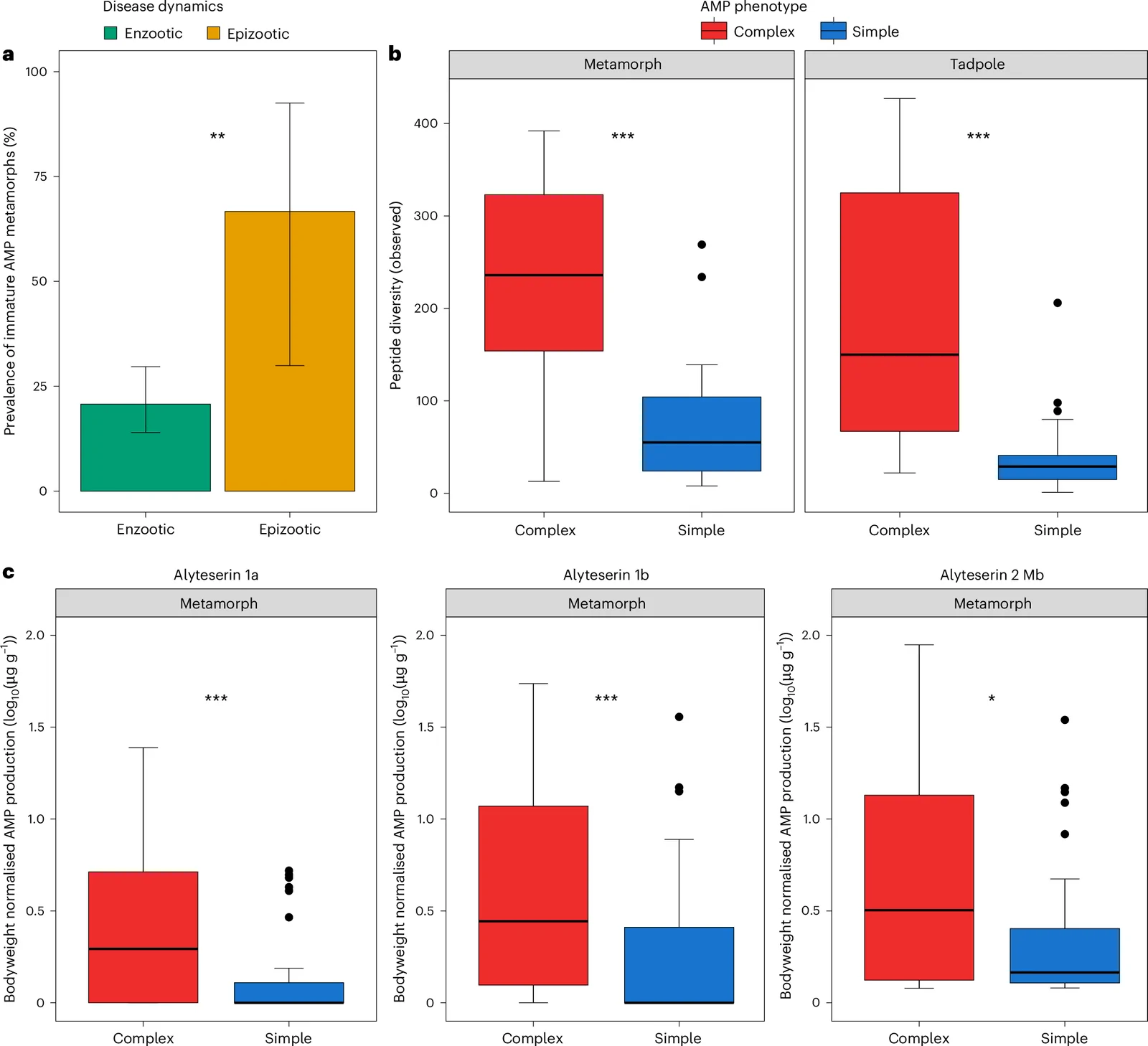
Timing of immune maturation is associated with *Bd* disease dynamics. **a**, Bar chart showing a significantly higher relative abundance of metamorphs with an immature AMP phenotype at the epizootic Lac d’Arlet (*N* = 9) population compared to the three enzootic populations (*N* = 114). Significance was determined using a Fisher’s exact test (*P* = 0.007). **b**, Box plot showing the significantly higher diversity of alyteserin-aligned peptides in animals with a mature (cluster 1) AMP phenotype (larvae, *n* = 61; postmetamorphic, *n* = 93) than immature (cluster 2) AMP phenotypes (larvae, *n* = 93; postmetamorphic, *n* = 30) across both life stages. Significance was determined using a two-sided Wilcoxon rank-sum test for larval (*W* = 5186, *P* < 0.001) and postmetamorphic (*W* = 2502, *P* < 0.001) life stages. **c**, Box plots showing the significantly higher bodyweight-normalized quantity of the three anti-*Bd* Alyteserins in recent metamorphs with a mature AMP phenotype (*n* = 93) than an immature AMP phenotype (*n* = 30). Significance was tested using an asymptotic Wilcoxon–Mann–Whitney test individually for alyteserin 1a (*Z* = 3.56, *P* < 0.001), alyteserin 1b (*Z* = 3.36, *P* < 0.001) and alyteserin 2 Mb (*Z* = 2.46, *P* = 0.013). All individuals represent biological replicates. Box plots show the median (center), 25th and 75th percentiles (box limits) and 1.5× the interquartile range (whiskers). Significance is denoted by *P < 0.05, **P < 0.01 and ***P < 0.001. Source data

The proportion of postmetamorphic individuals with a simple AMP phenotype was significantly higher (Fisher’s exact test: *P* = 0.007) in the epizootic population than the enzootic populations (Fig. 6a). We then wanted to test whether the lower alpha diversity of alyteserin-PVs in postmetamorphic animals at Lac d’Arlet (Fig. 4) was explained by this over-representation of simple AMP phenotypes.

We fitted a mixed-effect model of observed peptide diversity with cluster membership as an interacting effect, bodyweight and life stage as fixed effects and site as a random effect. We found that cluster membership was a strong predictor of observed peptide diversity (GlmmTMB: estimate = −152.68, s.e. = 11.85, *z* = −12.89, *P* < 0.001). Bodyweight (GlmmTMB: Est = 47.44, s.e. = 27.17, *z* = 1.75, *P* > 0.05) and life stage (GlmmTMB: Est = −32.08, s.e. = 17.69, *z* = −1.81, *P* > 0.05) were marginally nonsignificant. Across both life stages, complex AMP phenotypes possessed significantly higher alyteserin-PV alpha diversity than simple AMP phenotypes (Fig. 6b). Lastly, postmetamorphic animals with simple AMP phenotypes produced significantly smaller (bodyweight-normalized) quantities of *Bd*-inhibitory alyteserin 1a (Wilcoxon test: *Z* = 3.56, *P* < 0.001), alyteserin 1b (Wilcoxon test: *Z* = 3.36, *P* < 0.001) and alyteserin 2 Mb (Wilcoxon test: *Z* = 2.46, *P* = 0.013) (Fig. 6c).

## Discussion

This study challenges the paradigm of immune maturation being a fixed process through the ontogeny of organisms with a biphasic life history. This maturation process was presumed to be a transition from an immature (‘simple’) larval AMP phenotype to a mature (‘complex’) postmetamorphic AMP phenotype. We show that the timing of innate immune maturation relative to metamorphosis is associated with population-level disease dynamics in *A*. *obstetricans*. Animals that metamorphose before AMP immune maturity display a simple, low-diversity alyteserin-PV phenotype deficient in *Bd*-inhibitory AMPs. Study sites that have perennially declined following the emergence of *Bd* are characterized by a high prevalence of *A*. *obstetricans* manifesting this simple, low-diversity alyteserin-PV phenotype.

We show that the timing of immune maturation of amphibians is not intrinsically tied to metamorphosis. We further show that immune maturation may occur before metamorphosis and metamorphosis may occur without immune maturation. However, little is known about the factors that determine this timing. Whilst we acknowledge that a genetic component of this variation is likely, larval development in amphibians is known to be a highly plastic process. A wide range of interacting ecological factors experienced at the larval stage such as temperature, larval density, the presence of predators and food availability can influence many host traits, including skin chemistry. Although we are unable to produce definitive evidence to support our hypothesis, we believe that the process of larval overwintering in *A*. *obstetricans* likely explains this variation in immune investment before metamorphosis. Rather unusually for amphibians, *A*. *obstetricans* tadpoles can either achieve metamorphosis within a single year or potentially overwinter for one or more years before metamorphosis^45^. Overwintering is more common in high-elevation populations of *A*. *obstetricans*, likely because of constraints on the annual period of activity by the lower temperatures in montane habitats. This lack of overwintering and the shorter annual activity periods may leave tadpoles with insufficient resources to invest in immune development; accordingly, they metamorphose without mature AMP defenses. Our results highlight the importance of studying both amphibian life stages to understand population-level disease dynamics.

Through the application of MS-based peptidomics, we revealed that the diversity of secreted peptides from amphibian skin is far higher than was previously appreciated^32^. We found that 45% of these peptides had significant alignment with six known alyteserin AMPs. Interestingly, much of this variation appears to be driven by post-translational modifications. This includes the characterization of both C-terminal acid and C-terminal amide analogs of all six Alyteserin AMPs in these populations, modifications known to be functionally important in membrane interactions with bacteria^46^. The remaining modifications were likely driven by hydrolysis of mature AMPs and variation in the cleavage sites of mature AMPs from their protein precursor.

This increased complexity of amphibian AMP phenotypes also has broad implications for the study of immunity and eco-evolutionary theory. The innate immune system has long been thought of as a primitive, blunt instrument comprising a limited number of broad-spectrum antimicrobial effectors. However, this assumption is increasingly recognized as being inaccurate^5,8^. We demonstrated that alyteserins show antimicrobial selectivity in their inhibitory activity and post-translational modifications are driving a vast diversification of the peptidome from the transcribed proteome. However, despite these insights, we are currently unable to assess whether skin AMPs represent an inducible or constitutive defense against *Bd*. While *Bd* infection was a poor predictor of the overall composition of alyteserin-PVs, further work under controlled, laboratory conditions will be needed to assess whether individual AMPs are modulated in response to *Bd* infection.

The implications for this increase in characterized peptide diversity are complex. Currently, our ability to characterize these complex biological mixtures far exceeds our ability to understand their function. Machine-learning tools are now available that aim to predict the conformation^47^ or function^42^ of peptides from amino acid sequence. However, the current tools come with three primary limitations with respect to this study system. Firstly, many of these tools treat antimicrobial activity as a binary response variable, which ignores the nuances of the antimicrobial selectivity of host AMPs. All six known alyteserin AMPs found within this study are reported to show inhibitory activity to bacteria^32^ while only three of these peptides inhibit the growth of *Bd* in vitro. Secondly, the predicted tertiary structures and physiochemical characteristics derived from amino acid sequence are an integral part of predicting AMP activity (Supplementary Fig. 1). These properties are optimized for identifying AMPs that will interact with bacterial membranes, not the intracellular fungal inhibition demonstrated in this study. Further work is needed to understand the physiochemical properties of AMPs that inhibit fungi and how fungal pathogens are targeted by AMPs rather than the host’s epithelial cells. The traditional models of AMP activity show that initial interactions take places by electrostatic attraction between cationic AMPs and negatively charged phospholipid head groups of bacteria^48^. However, the keratinized tissue in which *Bd* encysts is also negatively charged^49^. A greater understanding of the properties of the membranes of amphibian epithelial cells and *Bd* will greatly improve our ability to perform in silico screenings of putative AMPs from large peptidomic datasets. Lastly, these tools assume ‘independence of action’ of AMPs. Although this is currently an unreasonable expectation of machine-learning tools, AMPs are known to interact with pathogens synergistically^33,34^ and ‘noninhibitory’ AMPs may act synergistically with other peptides to inhibit pathogen growth. However, with the rapid developments in this field, our ability to generate in silico predictions is constantly improving and future studies will be able to start to overcome these barriers.

The low alyteserin-PV diversity of the epizootic Lac d’Arlet population relative to the enzootic populations mirrors previous work with skin microbiomes in these populations^2,4^. The skin microbiome acts as extended immunity through the inhibition of *Bd* growth as a function of the production of anti-*Bd* metabolites^17^. These mucosal microbial communities are shaped and regulated through host AMP production^50^. However, in many epithelial systems, there is extensive bidirectional crosstalk between the host’s innate immunity and the microbiome^51^. This can include host immune priming modulated by the secretome of the skin microbiome when confronted by pathogens^52^. Future work should address these parallel observations through an integrative ‘holobiont’ approach to skin mucosal immunity.

Our results show that even intrinsically susceptible amphibian species can possess the tools that allow populations to recover following a severe *Bd* epizootic outbreak. In this study, we show that the timing of innate immune maturation relative to metamorphosis is associated with population-level disease dynamics in *A*. *obstetricans*. Animals that metamorphose before AMP immune maturity display a low-diversity AMP phenotype deficient in anti-*Bd* AMPs. A high incidence of these postmetamorphic animals with immature AMPs is associated with epizootic disease dynamics and population decline. These observed differences in host immune phenotype development are likely a crucial mechanism for establishing stable host–pathogen coexistence. As these immune phenotypes and biphasic history are highly generalizable across the class Amphibia, our results suggest that the timing of innate immune maturation may underlie the observed rebound in other *Bd*-susceptible amphibian species.

## Methods

### Sample collection: field surveys

Field sampling took place between July 29 and August 6, 2019, and between August 3 and August 9, 2020. Animals were captured by hand or net and we sampled a maximum of 20 tadpoles and 20 postmetamorphic individuals per site (Supplementary Table 3). Recently metamorphosed individuals were found beneath cover objects bordering the lakes and any observed mortalities were recorded. These postmetamorphic animals only congregate near water for a few weeks before dispersing into strictly terrestrial habitats, which limits demographic structuring of the animals sampled^45^. Tadpoles had their mouthparts swabbed with an MW100 cotton-tipped swab (Medical Wire and Equipment) and postmetamorphic animals were swabbed according to a standard protocol; each specimen was swabbed by taking five strokes down the center of the underside, on each flank, the inside of the hind legs and bottom of the feet. Swabs were stored below 4 °C until DNA extraction. Peptide sampling was performed using a bath exposure of norepinephrine^53^. Briefly, each individual was exposed to a 200 μM bath of norepinephrine bitartrate (A0397, Merck) and high-performance LC (HPLC)-grade water (V270733, Merck) in a sterile Whirlpak bag (Nasco Sampling). After 15 min, the animal was removed from the Whirlpak and returned to its individual capture bag. The sample was then acidified by addition of 0.2 ml of trifluoroacetic acid (302031, Merck), returned from the field on ice and stored at −18 °C until purification for MS analysis. Each animal was contained in a new plastic bag and handled with a new pair of powder nitrile gloves to prevent cross-infection of individuals^54^.

### DNA extraction and *Bd* diagnostics

DNA from skin swabs was extracted in a PrepMan Ultra (Applied Biosystems). To assign the infection status and to quantify the disease burden of each individual, we performed *Bd*-ITS qPCR^55^. Extractions were diluted 1:10 before qPCR amplification, run in duplicate and considered to be positive if both wells amplified and were over 0.1 genomic equivalents (GE; where 1 GE is the quantity of genetic material present in a single zoospore). All samples were run against positive standards of 0.1, 1, 10 and 100 GE of *Bd-*GPL (isolate IA042) and negative control standards. Plates with a standard curve of *R*^2^ < 0.95 were repeated.

### Skin secretion sample prep

Skin secretion samples were concentrated and desalted through a C18 Sep-Pak cartridge (WAT020515, Waters) partial purification procedure^53^. Briefly, the Sep-Pak cartridge was primed with 2 ml of acetonitrile (270717, Merck), equilibrated with 2 ml of a 0.1% TFA wash solution before the sample was loaded. To maximize the yield, the wash and loading steps were repeated. Samples were then stored on the cartridges at 4 °C until analysis. Before MS analysis, the cartridge was washed with 4 ml of a 0.1% TFA wash solution before being eluted with 2 ml of 70% acetonitrile and 30% HPLC water with 0.1% TFA. The eluent was then frozen overnight at −80 °C and dried under vacuum using a Speedvac (Thermo-Savant).

### LC–MS/MS analysis of field samples

Proteomic analysis of the semipurified amphibian skin secretions was performed using a Vanquisher LC system connected to a Q-Exactive plus quadrupole-orbitrap MS instrument (Thermo Scientific). Lyophilized skin secretions were resuspended in 100 μl of solvent (5% acetonitrile in water, 0.1% formic acid) and centrifuged for 5 min at 11,300*g*. Then, 20 μl of each sample was injected onto a Hypersil Gold column (150 mm × 2.1 mm, 3-µm particle size; 25002-1521300). The chromatographic peptides separation on this column was achieved using mobile phase A (water, 0.1% formic acid) and mobile phase B (acetonitrile, 0.1% formic acid). The 20-min gradient was as follows: 2 min with 5% B, before increasing to 99% B over the next 15 min, maintaining at 99% B for a further 3 min and then decreasing to 5% B within 6 s, maintained for 1.9 min to equilibrate the Hypersil C_18_ column. The flow rate was 0.2 ml min^−1^. The column effluent was continuously directed to the heated electrospray ionization (HESI) source of the Q-Exactive MS instrument. The HESI probe was operated with sheath gas flow at 25, auxiliary gas flow at 5, spray voltage at 3.80, capillary temperature at 270 °C, S-lens radiofrequency level at 55 and auxiliary gas heater temperature at 50 °C. The Q-Exactive MS instrument was operated in a positive ion mode. MS data were acquired using a data-dependent acquisition mode and operated at 70,000 resolution (full width at half-maximum height definition). The top eight most abundant multiply charged (*z* ≥ 2) ions in the 300–1,800 *m*/*z* range were selected for MS/MS, with dynamic exclusion enabled for 30 s. The automatic gain control for the Q-Exactive was set to 500,000 ions and the automatic gain control for the MS/MS in the ion trap was set at 8,000 ions. For MS/MS, the isolation width was set at 1.4 and the collision energy was 35%. MS/MS scans were acquired at a resolution of 17,500 at 200 *m*/*z* and the fixed first mass was set to 120 *m*/*z*. Three MS/MS microscans for each precursor were accumulated. Dynamic exclusion was enabled and selected ions were excluded for 20 s before they could be selected for another round of MS/MS.

### Peptidomic reference libraries

To align the peptide sequences generated through MS, we used two references libraries. To identify known AMPs, we aligned all de novo sequences against the APD3 database^39^.

To generate a full mucosal peptidome, we generated an RNA-seq reference library for both larval and postmetamorphic *A*. *obstetricans*. Skin samples (of three larval and three postmetamorphic *A*. *obstetricans*) up to 25 mg were collected and immediately stored in Trizol reagent. Samples were homogenized using a pestle in 1.5-ml tubes and total RNA was extracted using the standard Trizol RNA extraction protocol. Pelleted RNA was resuspended in 30 µl of RNase-free water. RNA integrity was quantified using an Agilent TapeStation 2100 bioanalyzer. Total RNA was quantified on a Qubit using the RNA high-sensitivity kit. Sample replicates were pooled equally to produce a single sample for library preparation. Samples were submitted to Novogene for mRNA library preparation (poly(A) enrichment) and paired-end 150-bp sequencing on an Illumina NovaSeq for 80 million reads per sample.

De novo assembly was performed using Trinity (version 2.8.5) using default parameters. Trimmomatic (version 0.39)^56^ was used to quality trim reads and in silico normalization of reads was carried out. Resulting assemblies were quality assessed by examining the number of transcripts that appear full-length or nearly full-length. BLASTx (version 2.2.30), using the *Xenopis* *laevis* (XENLA_10.01_GCF_XBmodels.transcripts.fa) reference transcript set as a frog-specific comparison, was performed to assess the quality of the assemblies. It is sometimes the case that a single transcript will align to a single protein sequence with several discontinuous alignments (that is, a BLAST hit containing multiple hit-scoring segment pairs (HSPs)). Therefore, multiple HSPs were grouped per transcript and database hit and the alignment coverage based on grouped HSPs was also assessed. Bowtie2 (version 7.3.0)^57^ was used to assess the alignment rate of RNA-seq reads back to the assembly. The two assemblies were then concatenated and underwent six-frame translation in EMBOSS sixpack^58^ before being clustered into 95% similar sequences using CD-HIT^59^.

### Peptidomic pipeline

Downstream analysis of the MS data was conducted using PEAKs Studio (version 12.0; Bioinformatic Solutions). From 697,265 MS scans and 1,109,355 MS/MS scans, 1,073,825 peptide spectrum matches were identified using the DeepNovo algorithm^40^ with the following parameters: no enzyme digestion, parent mass error tolerance of 10.0 ppm, fragment mass error tolerance of 0.02 Da and a maximum of three post-translational modifications per peptide. We then performed a two-round search of de novo sequences with a quality score > 70 against APD3 and then our transcriptome-derived *A*. *obstetricans* peptide library with an identification threshold of −log*P* > 20. Lastly, high-quality unaligned sequences were assembled into de novo peptide features. Label-free quantification using PEAKs Q (version 12.0; Bioinformatic Solutions) was used to quantify the expression levels of the peptides in each sample using the following parameters: retention time, 4–22 min; peptide quality, ≥5; peptide average area, ≥1 × 10^6^; peptide charge, 1–5; protein significance, ≥20; fold change, ≥1.

### AMP quantification

Relative quantification of AMPs was achieved through generating a standard curve using synthetic peptides supplied by Cambridge Research Biochemicals. Standards were made up at concentrations of 100 μM, 50 μM, 25 μM and 12.5 μM in 5% acetonitrile and 95% water with 0.1% formic acid using the same LC–MS method used for the amphibian secretion samples. Peak areas of the standards were measured using Xcalibur Qual browser (version 2.2; Thermo Scientific). All standard curves had an *R*^2^ > 0.95.

### In vitro *Bd* inhibition assays

Growth inhibition assays were conducted by challenging *Bd-*GPL343 with a dilution series (six replicates of 100 μM, 50 μM, 25 μM, 12.5 μM, 6.25 μM, 3.125 μM and 1.60 μM) of each AMP in a FLUOstar Omega optical density plate reader (BMG Labtech). The experimental set up included a positive control (50 μl of *Bd* and 50 μl of sterile water), negative control (50 μl of heat-killed *Bd* and 50 μl of sterile water) and blanks (50 μl of mTGHL medium and water)^60^. Blank-compensated optical density readings were taken daily and each well examined with a Leica inverted microscope to check for contamination. Contaminated wells were removed from the experiment. Readings on each plate were taken until the growth rate of the positive control had reached a plateau. Growth was measured as a percentage of the mean growth of the positive control wells on the day growth reached a plateau. Best fit log–log models were determined and fitted using R package DRC^61^.

### Confocal microscopy imaging of Cy3-labeled alyteserins with *Bd*

We challenged killed *Bd*-GPL isolate IA042 against Cy3-labeled alyteserin 1a, alyteserin 2c and scr-alyteserin 2c (Supplementary Document 1). Briefly, zoospores of *Bd*-GPLIA042 were isolated from a 3-day old culture by filtration of the mTGhL on a 10-µm filter. Then, 300 µl of the zoospore suspension were inoculated in Mattek petri dishes for 2 days at 17 °C. Young zoosporangia were then killed and blocked by replacing the medium with a solution of 0.5% sodium azide and 1× carbofree blocking solution (Vectorlabs) in 1× PBS and incubating at 37 °C for 15 min. As dead zoosporangia were used, the potential bias raised by the different biological activities of the alyteserins was alleviated and all zoosporangia could be stained with a similar concentration of alyteserins and fluorophores. Blocked zoosporangia were then incubated with 10 µM Cy3–alyteserins in 1× PBS overnight at 4 °C and counterstained with 10 µg ml^−1^ calcofluor white for 15 min. After two washes, zoosporangia were imaged on Zeiss LSM780 laser scanning confocal microscope accumulating *Z* stacks covering the entire organism with a ×63 objective.

### Statistical methods

All statistical analyses were carried out in R (version 4.2.0)^62^. Proteomic data were handled in the R package phyloseq^63^ and CLR data transformations were performed in the R package microbiome. Curve fitting of peptide inhibition data was performed in the R package DRC. All plots were generated using ggplot2 (ref. ^64^). The map of field sites was generated using the ESRI National Geographic (https://services.arcgisonline.com/ArcGIS/rest/services/NatGeo_World_Map/MapServer) and ESRI Gray (light; https://www.arcgis.com/home/item.html?id=8b3d38c0819547faa83f7b7aca80bd76) base layers in QGIS^65^.

### Reporting summary

Further information on research design is available in the Nature Portfolio Reporting Summary linked to this article.

## Online content

Any methods, additional references, Nature Portfolio reporting summaries, source data, extended data, supplementary information, acknowledgements, peer review information; details of author contributions and competing interests; and statements of data and code availability are available at https://doi.org/10.1038/s41589-026-02254-6.

## Supplementary information

Supplementary Information Supplementary Tables 1–3, Fig. 1 and Documents 1–3.

Reporting Summary

## Source data

Source Data Fig. 1 Statistical source data.

Source Data Fig. 2 Statistical source data.

Source Data Fig. 2 Statistical source data.

Source Data Fig. 4 Statistical source data.

Source Data Fig. 5 Statistical source data.

Source Data Fig. 6 Statistical source data.

## Data Availability

The MS proteomics data were deposited to the ProteomeXchange Consortium through the PRIDE partner repository with the dataset identifier PXD069814. All raw RNA reads were deposited to the European Bioinformatics Institute European Nucleotide Archive and National Center for Biotechnology Information Sequence Read Archive under project accession PRJEB95135. Skin transcriptome assemblies for the postmetamorphic and larval *A*. *obstetricans* were deposited to Zenodo (https://doi.org/10.5281/zenodo.17463202 and https://doi.org/10.5281/zenodo.17463248)^66,67^. All source data are available from DataDryad (https://doi.org/10.5061/dryad.k0p2ngfn6)^68^. Source data are provided with this paper.

## Code availability

All R scripts are available from DataDryad (https://doi.org/10.5061/dryad.k0p2ngfn6)^68^.
