# Supplementary material for: Early maturation of host antimicrobial peptide defences is associated with host–pathogen coexistence
Source: Nat Chem Biol. 2026 Jul 15;22(11):1731–40. doi: 10.1038/s41589-026-02254-6 (PMC13710165; doi:10.1038/s41589-026-02254-6)
Supplement: Supplementary file 1 [file 41589_2026_2254_MOESM1_ESM.pdf]

nature chemical biology

Supplementary information

https://doi.org/10.1038/s41589-026-02254-6

Early maturation of host antimicrobial peptide defences is associated with host–pathogen coexistence

In the format provided by the authors and unedited

Supplementary information

Peptide	Sequence	Reference	α-helical?
Alyteserin-1a	GLKDIFKAGLGSLVKGIAAHVAN-NH2	Conlon et al. 2009	Yes
Alyteserin-1b	GLKEIFKAGLGSLVKGIAAHVAN-NH2	Conlon et al. 2009	Yes
Alyteserin-1c	GLKEIFKAGLGSLVKGIAAHVAS-NH2	Conlon et al. 2009	Yes
Alyteserin-1d	GLKDIFKAGLGSLVKNIAAHVAN-NH2	Conlon et al. 2009	Yes
Alyteserin-2a	ILGKLLSTAAGLLSNL-NH2	Conlon et al. 2009	Yes
Alyteserin-2Mb	ILGAIIPLVSGLLSHL-NH2	König et al. 2012	Yes

Table S1 Table showing previously described Alyteserin AMPs found in this study. Predicted structures for these AMPs were generated in PEP-FOLD3 and shown in Fig.S1

Peptide	Model type	Hill slope (Estimate)	Hill slope (S.E)	Min value (Estimate)	Min value (S.E)	Max value (Estimate)	Max value (S.E.)	EC50 (Estimate)	EC50 (S.E.)
**Alyteserin-1a**	**LL4**	**-4.65**	**1.65**	**14.59**	**4.67**	**93.05**	**3.88**	**8.47**	**0.95**
**Alyteserin-1b**	**LL3**	**-0.94**	**0.45**	**0**	**0**	**107.69**	**66.13**	**41.03**	**60.57**
**Alyteserin-1c**	**N/A**								
**Alyteserin-1d**	**N/A**								
**Alyteserin2a**	**N/A**								
**Alyteserin-2b**	**N/A**								
**Alyteserin-2c**	**LL4**	**-1.78**	**0.92**	**15.63**	**6.65**	**104.3**	**7.98**	**37.14**	**7.4**
**Alyteserin-2Mb**	**LL3**	**-4.65**	**1.2**	**0**	**0**	**100.34**	**3.06**	**7.53**	**0.45**

Table S2 *In-vitro* assay results against *Bd* showing the best fit models for the inhibition curves of each peptide.

Site	Year	Life stage	Swabs	Peptide
Lac d'Arlet	2019	Tadpole	23	23
		Metamorph	2	2
	2020	Tadpole	20	20
		Metamorph	7	7
Puits d'Arious	2019	Tadpole	20	19
		Metamorph	20	17
	2020	Tadpole	20	15
		Metamorph	20	20
Lac de Lhurs	2019	Tadpole	20	20
		Metamorph	20	18
	2020	Tadpole	20	20
		Metamorph	20	19
Ibón de Acherito	2019	Tadpole	20	18
		Metamorph	20	20
	2020	Tadpole	20	19
		Metamorph	20	20

Table S3 Sample table showing the number of *A. obstetricans* skin swab and peptide samples broken down by site, year and life stage

Supplementary figures

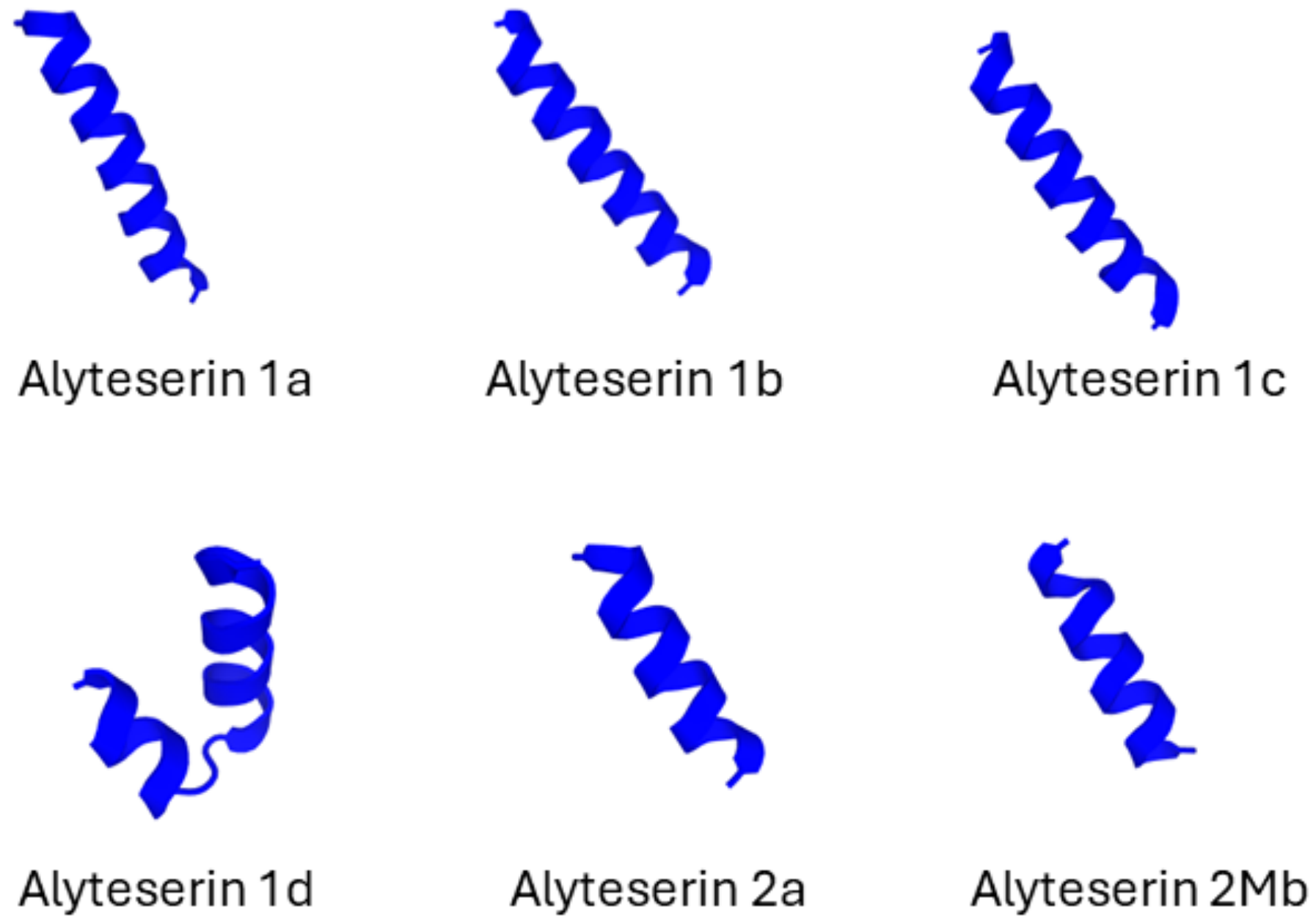

Figure S1 Predicted structures of Alyteserins found in the study populations were generated using PEP-FOLD3 under default settings

Document S1

Peptide synthesis

Alyteserin 1a	GLKDIFKAGLGSLVKGIAAHVAN-NH_2
Alyteserin 2c	ILGAILPLVSGLLSSKL-NH_2
scr-Alyteserin 2c	LGGLIKLVSASLPISLL-NH_2

All automated peptide synthesis was carried out using the MultiSynTech Syro peptide synthesiser. Fmoc-protected amino acids, solvents and resins were purchased from Merck.

Fmoc deprotection of resin/peptides: To Rink Amide Resin (100 mg), pre-swollen in DMF (1 x 2 mL), was added a solution of (40% v/v) piperidine in DMF (1.5 mL). The reaction was shaken for 3 min, and the DMF removed under vacuum. The resin was then washed with DMF (6 × 1.5 mL), and an additional equivalent of piperidine (20% v/v) in DMF (1.5 mL) was added to the reaction syringe, and the resin shaken for a further 10 mins. Following completion, the solution was removed under vacuum and the resin washed with DMF (6 ×1.5 mL).

Double coupling of Fmoc-protected amino acids: Fmoc-protected amino acid (3 eq.), HATU/HBTU (3 eq.) and DIPEA (6 eq.) were sequentially added to the deprotected resin, and the resin was agitated for a total of 40 min. The coupling reagents were then removed under vacuum filtration and an additional aliquot of the coupling solution was added. After which, the reaction was agitated for an additional 40 min and washed with DMF (4 × 1.5 mL). All peptides were double coupled, so the cycles of on resin coupling, agitation and washing were repeated twice.

Once synthesised, peptides were cleaved from the resin using a cleavage solution made of TFA (965 µL), water (25 µL), TIPS (10 µL). The resin was initially washed with CH_2Cl_2 (3 x 2 mL), MeOH (3 x 2 mL) and diethyl ether (3 x 2 mL) and dried under vacuum for 30 min. After this, the cleavage solution was prepared (as described above), added to the resin, and left to shake for 40 mins at

350 rpm. Once cleaved, the solution was expelled into a Falcon tube containing cold diethyl ether (7 mL), and the total volume of the solution was adjusted to 10 mL with diethyl ether. The solution was centrifuged for 25 min at (4000 rpm, 4 °C), which provided the peptide as a white solid. The ether was then decanted, and an additional aliquot of the cleavage solution (1 mL) was added to the resin, and the resin agitated for an additional 30 min. The cleavage solution was again directly evacuated into same falcon tube and the total volume was again adjusted to 10 mL with ether. The following solution was centrifuged for 25 mins at (4000 rpm, 4 °C), which provided the peptide as a white solid. The ether was then again decanted, the peptide pellet disturbed, re-dissolved in ether (8 mL) and centrifuged. These cycles of decanting, resuspending and centrifugation were repeated once more. Once complete, the residual ether was evaporated off and the crude peptide was re-suspended in water (10 mL) and lyophilised, ready for purification.

All peptides were purified by reverse phase HPLC using a Dionex 580 chromatography system, equipped with Aligent ZORBAX 300SB-C18 (9.4 x 250 mm) column, with UV detection at (214 – 254 nm) using the gradients reported. Analytical HPLC was performed using the Aligent 1260 Infinity II chromatography system, with detection at (214 nm), equipped with a ACE 5-C18-AR column (150 × 4.6 mm), using acetonitrile and water (0.1% v/v TFA) as the mobile phases. Peptides were lyophilised using the Vir Tis Benchtop Pro freeze dryer. LRMS were performed on the Waters Acquity UPLC SQD mass spectrometer, equipped with a C8 column using a gradient of 5-95% acetonitrile over 5 mins, with HPLC grade water (0.1% v/v formic acid) and acetonitrile (0.1% v/v formic acid) as the mobile phases. Spectral analysis was performed using Mass Lynx 4.1 software.

Alyteserin 1a, Alyteserin 2c and scr-Alyteserin 2c were synthesised via standard Fmoc solid-phase peptide synthesis using Rink amide resin, as above. Upon completion, the peptides were cleaved from the resin following standard cleavage protocols and purified by preparative HPLC.

Samples of Alyteserin 2c and scr-Alyteserin 2c were also custom synthesised by CanPeptide, Inc (St-Laurent, QC Canada).

Synthesis of Cy-3 labelled peptides for confocal experiments

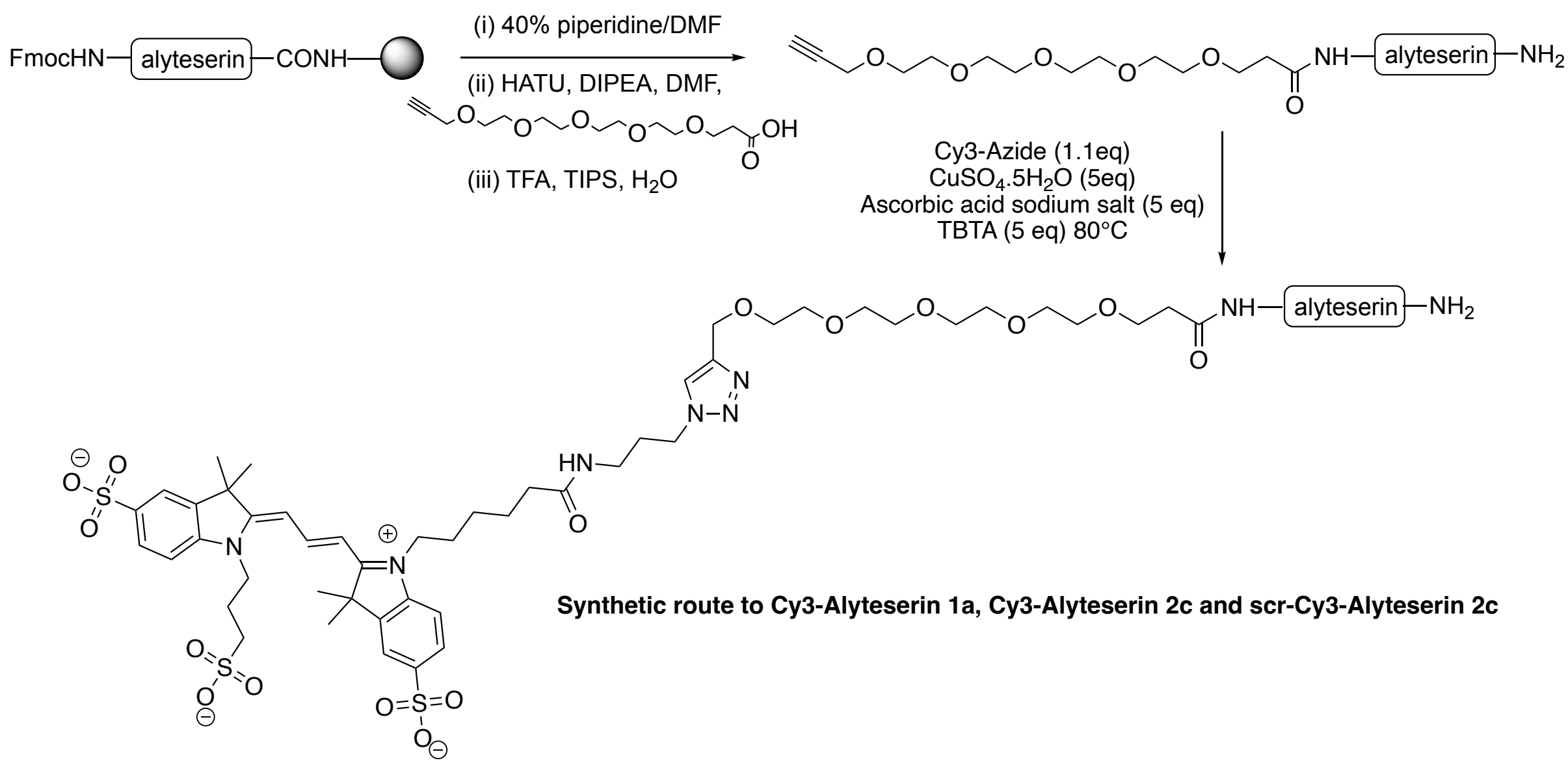

Synthetic route to Cy3-Alyteserin 1a, Cy3-Alyteserin 2c and scr-Cy3-Alyteserin 2c

The solid phase synthetic routes to Alyteserin 1a, Alyteserin 2c and scr-Alyteserin 2c were modified to give the required Cy3-labelled alyteserins as follows. Deprotection of the N-terminal Fmoc group of the resin-bound intermediate was followed by coupling of the commercially available alkyne-PEG5-acid (Merck, UK) using standard coupling conditions. The peptides were cleaved from the resin and purified via reverse phase HPLC as described above. The purified peptides were then fluorescently modified using click chemistry as described below.

Cy3-Alyteserin 1a: A solution of alkyne-PEG5-Alyteserin-1a (1 mg, 390 nmol) in DMF (50 µL) and Cy3 azide (0.3 mg, 390 nmol) in water (50 µL) was transferred to a 250 µL microwave vial. Stock solutions of $CuSO_4 \cdot 5H_2O$ (36.1 mg, 5.00 mL water), sodium ascorbate (58 mg, in 5.00 mL water) and tris[(1- benzyl-1H-1,2,3-triazol-4-yl)methyl]amine (TBTA) (0.94 mg, 500 µL DMF) were made up. 50.0 µL of each stock solution was added to the reaction vial. The reaction mixture was heated for 20 min at 80 °C. After completion, the reaction mixture was quenched with buffer (3 mL, 0.1%

TFA in water), filtered and purified by reverse phase analytical HPLC using ACE 5-C18-AR column (150 × 4.6 mm) with a gradient of 5–45% over 40 min at 1 mL min^{-1} with detection at 214 nm. LRMS: (ES+) $[C_{153}H_{241}N_{36}O_{43}S_3]^+$ calculated = 3368.3; found m/z = 1124.7 $[M+3H]^{3+}$, 843.6 $[M+4H]^{4+}$, 675.4 $[M+5H]^{5+}$.

Cy3-Alyteserin 2c: A solution of alkyne-PEG5-Alyteserin-2c (1 mg, 505 nmol) in DMF (50 µL) and Cy3 azide (0.4 mg, 505 nmol) in water (50 µL) was transferred to a 250 µL microwave vial. Stock solutions of $CuSO_4 \cdot 5H_2O$ (59 mg, 5.00 mL water), sodium ascorbate (95 mg, in 5.00 mL water) and tris[(1- benzyl-1H-1,2,3-triazol-4-yl)methyl]amine (TBTA) (1.54 mg, 500 µL DMF) were made up. 50.0 µL of each stock solution was added to the reaction vial. The click reactions were heated for 20 min at 80 °C. After completion, the reaction mixture was quenched with buffer (3 mL, 0.1% TFA in water), filtered and purified by reverse phase analytical HPLC using ACE 5-C18-AR column (150 × 4.6 mm) with a gradient of 5–45% over 30 min at 1 mL min^{-1} with detection at 553 nm. HRMS: (ES+) $[C_{129}H_{210}N_{25}O_{36}S_3]^+$ calculated = 2784.18 ; found m/z = 1393.2 $[M+2H]^{2+}$, 934.7 $[M+3H+H_2O]^{3+}$

Cy3-scr-Alyteserin 2c: A solution of alkyne-PEG5-scr-Alyteserin-2c (1 mg, 505 nmol) in DMF (50 µL) and Cy3 azide (0.4 mg, 505 nmol) in water (50 µL) was transferred to a 250 µL microwave vial. Stock solutions of $CuSO_4{\cdot}5H_2O$ (59 mg, 5.00 mL water), sodium ascorbate (95 mg, in 5.00 mL water) and tris[(1- benzyl-1H-1,2,3-triazol-4-yl)methyl]amine (TBTA) (1.54 mg, 500 µL DMF) were made up. 50.0 µL of each stock solution was added to the reaction vial. The reaction mixture was heated for 20 min at 80 °C. After completion, the reaction mixture was quenched with buffer (3 mL, 0.1% TFA in water), filtered and purified by reverse phase analytical HPLC using ACE 5-C18-AR column (150 × 4.6 mm), gradient of 5–45% over 30 min at 1 mL min^{-1} with detection at 553 nm. LRMS: (ES+) $[C_{129}H_{210}N_{25}O_{36}S_3]^+$ calculated = 2783.18; found m/z = 1393.6 $[M+2H]^{2+}$, 928.9 $[M+3H+H_2O]^{3+}$

Document S2

HPLC and mass spectral data on Cy3-labelled peptides

Cy3-Alyteserin 1a

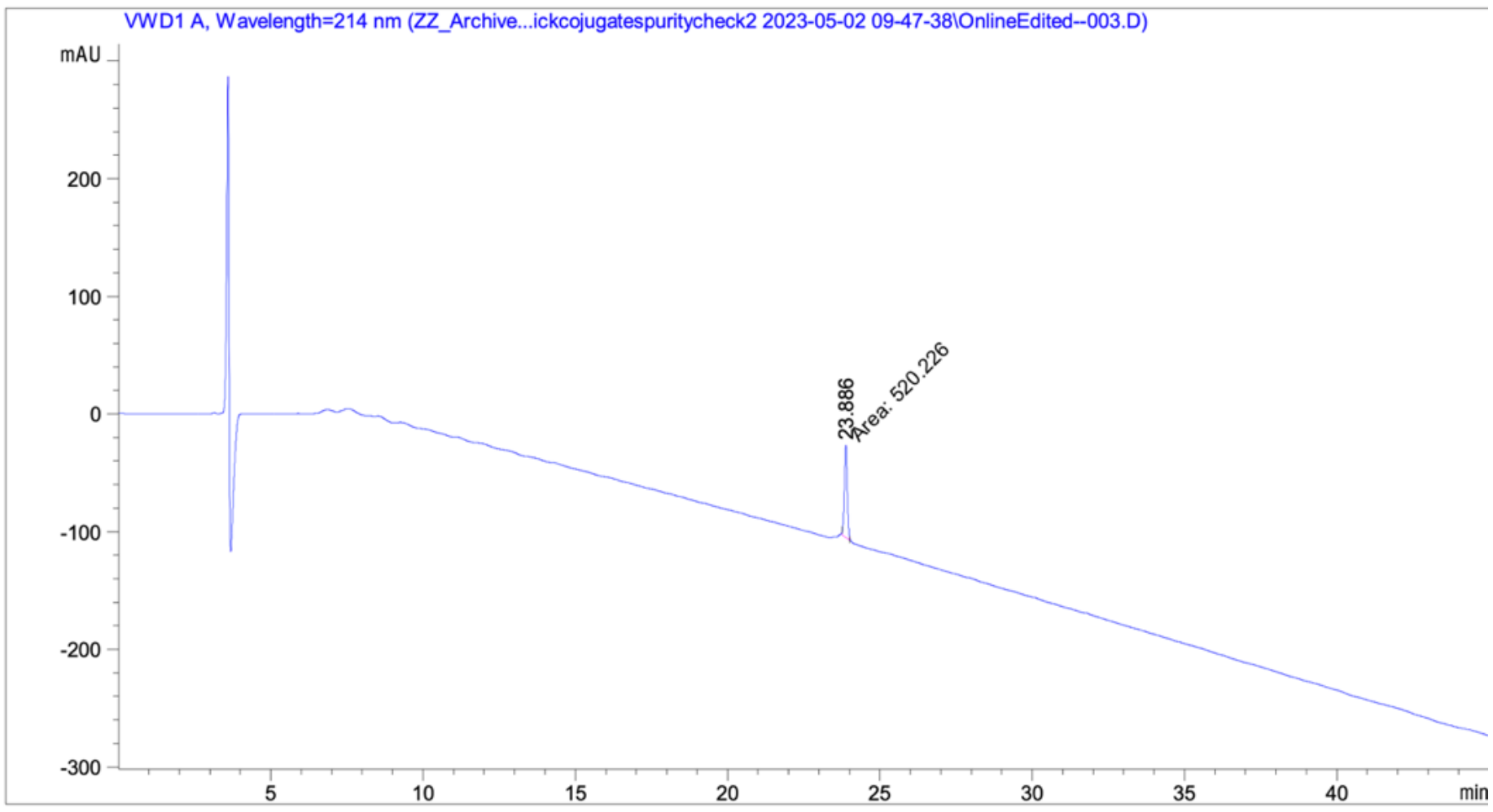

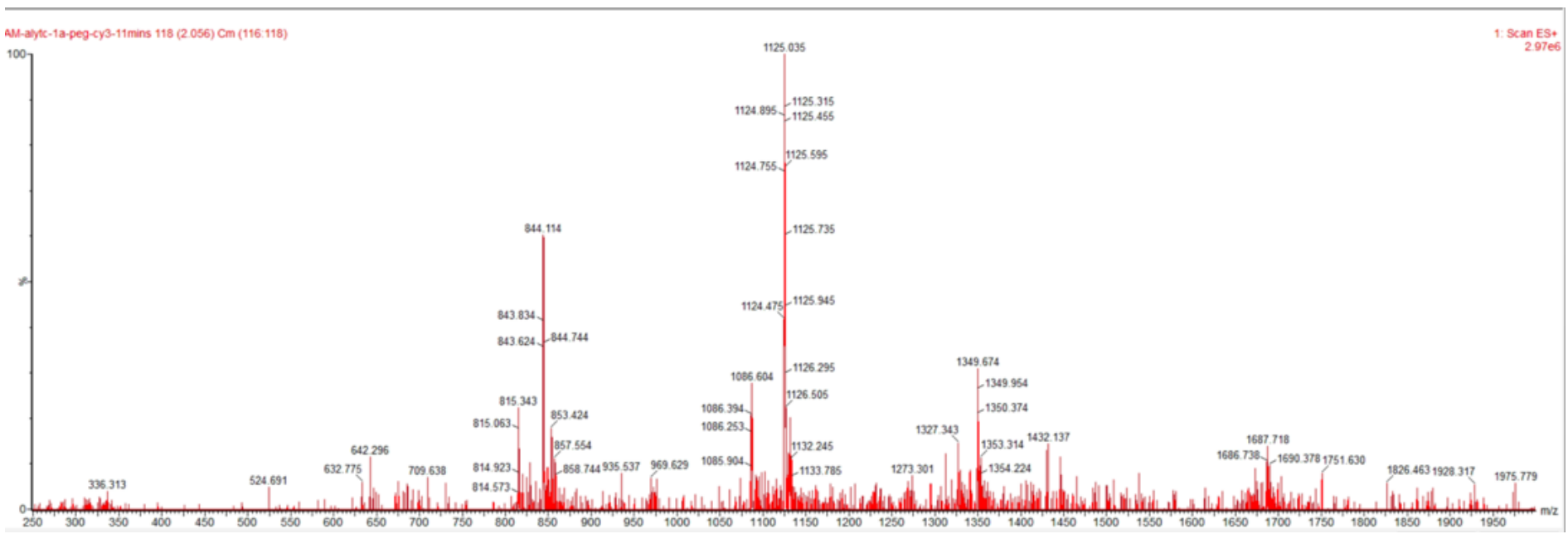

Cy3-Alyteserin 2c

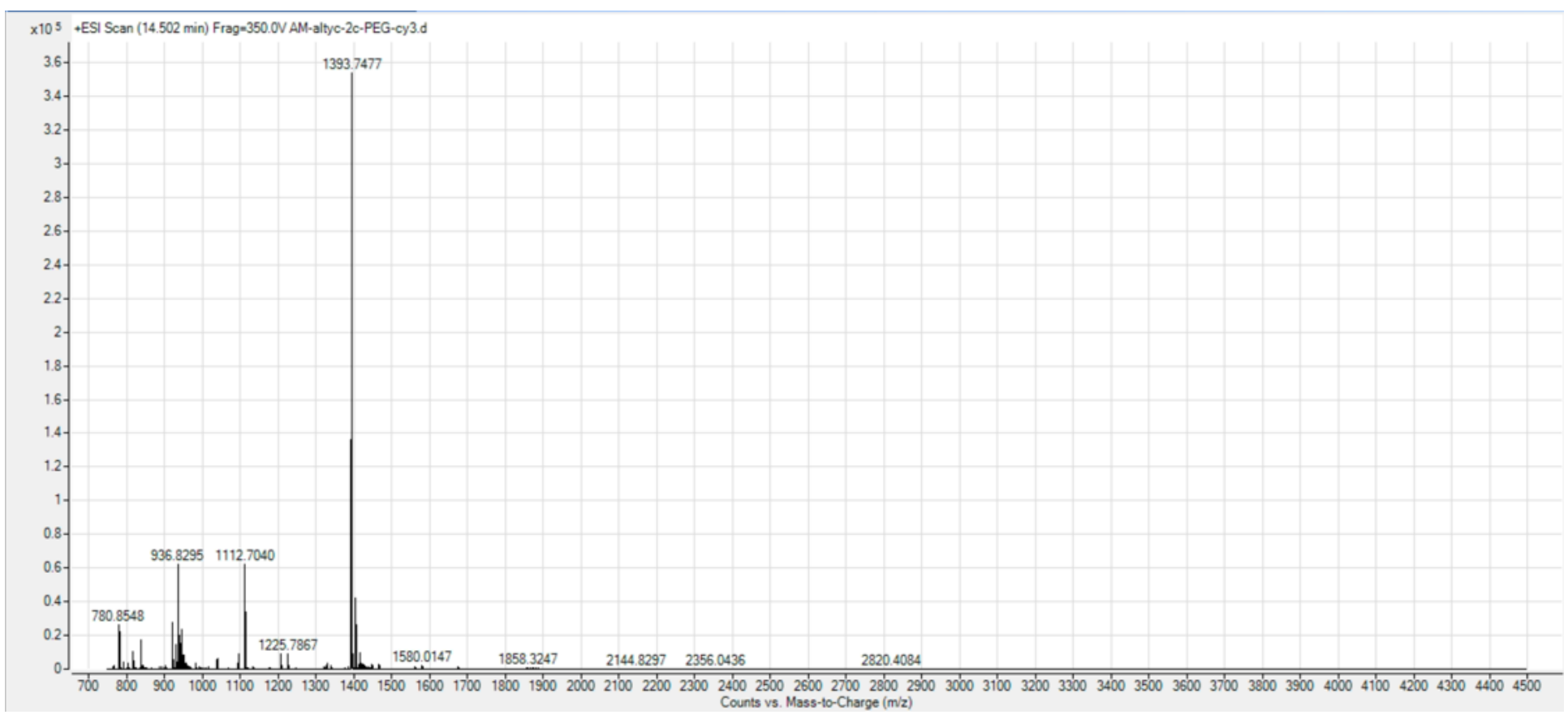

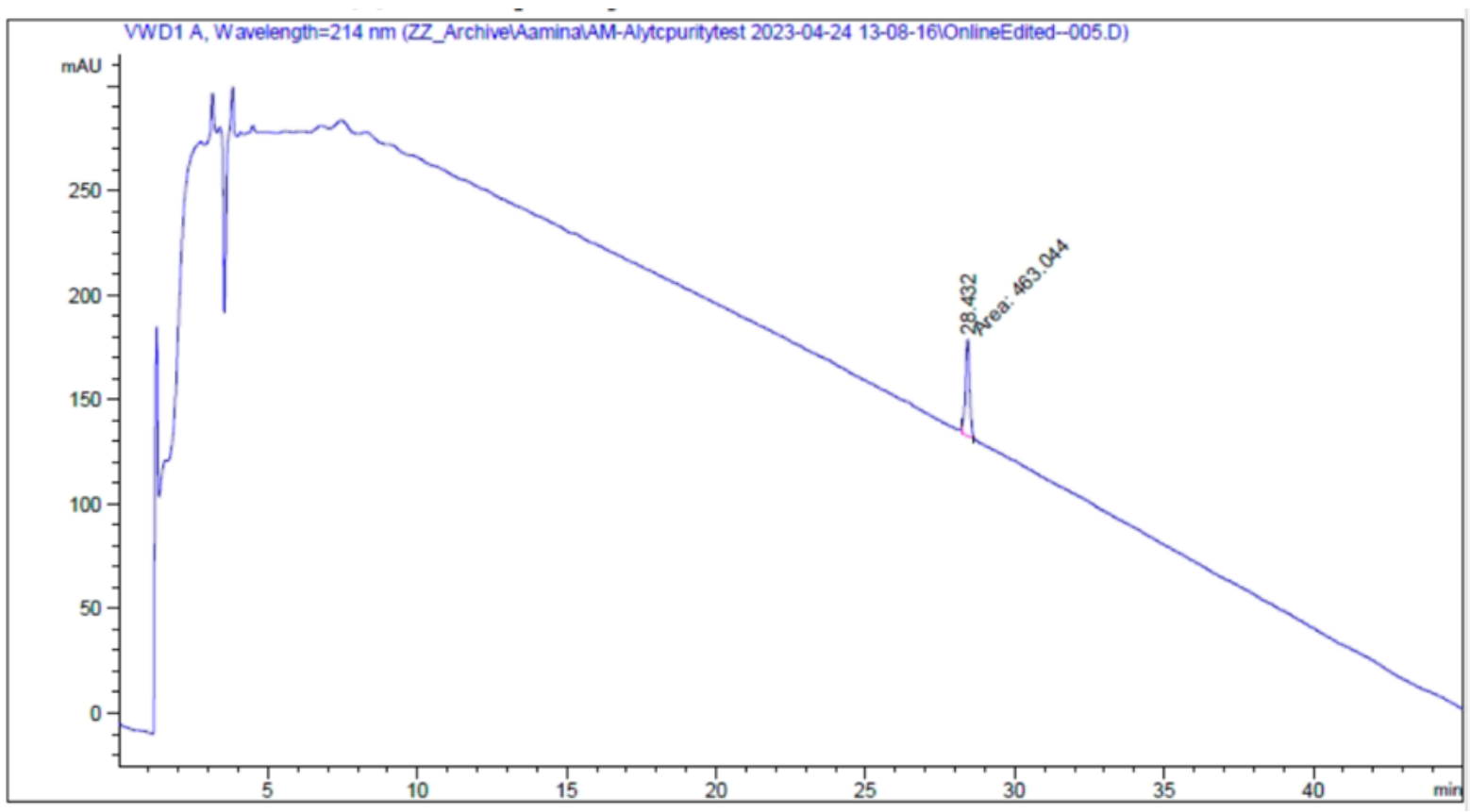

Cy3-scr-Alyteserin 2c

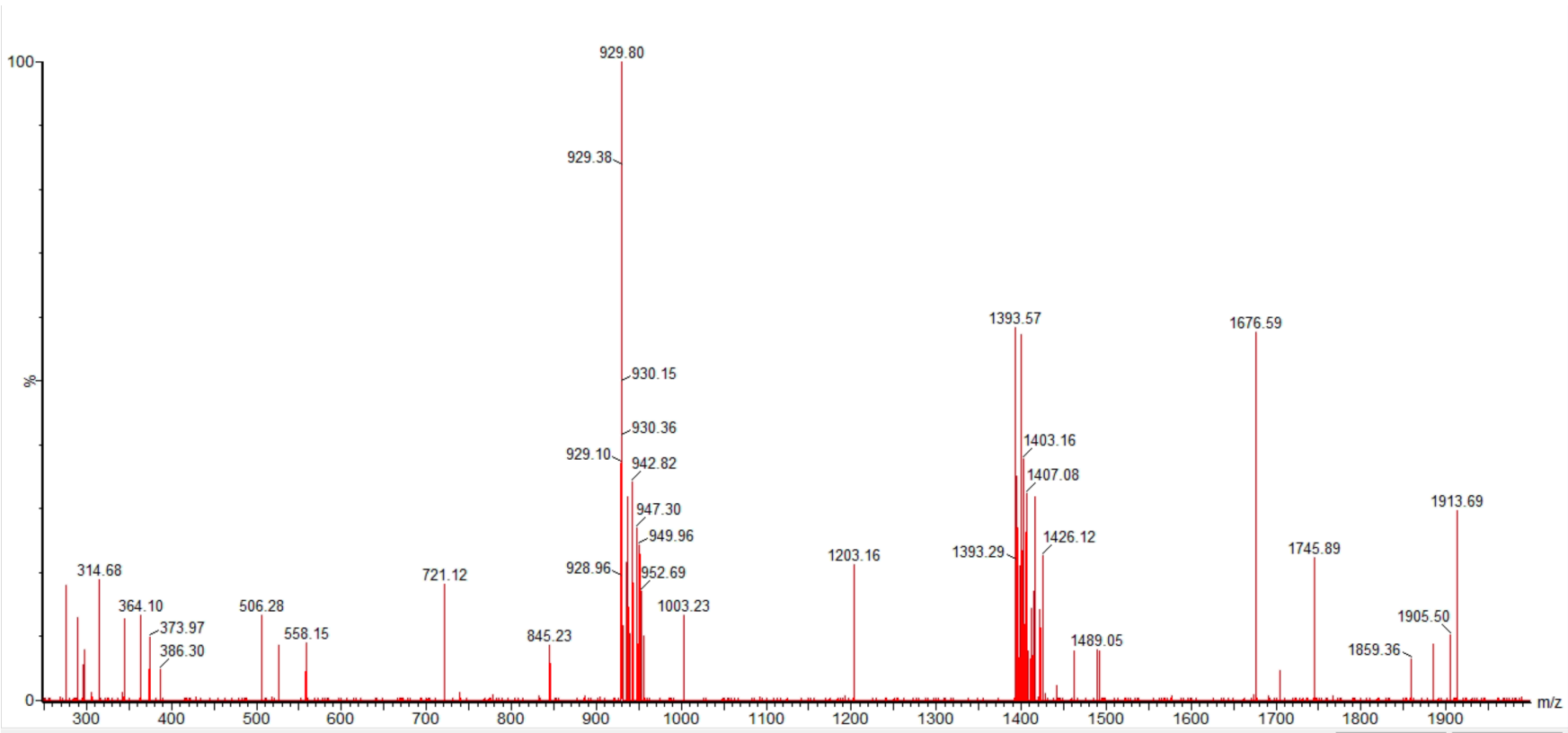

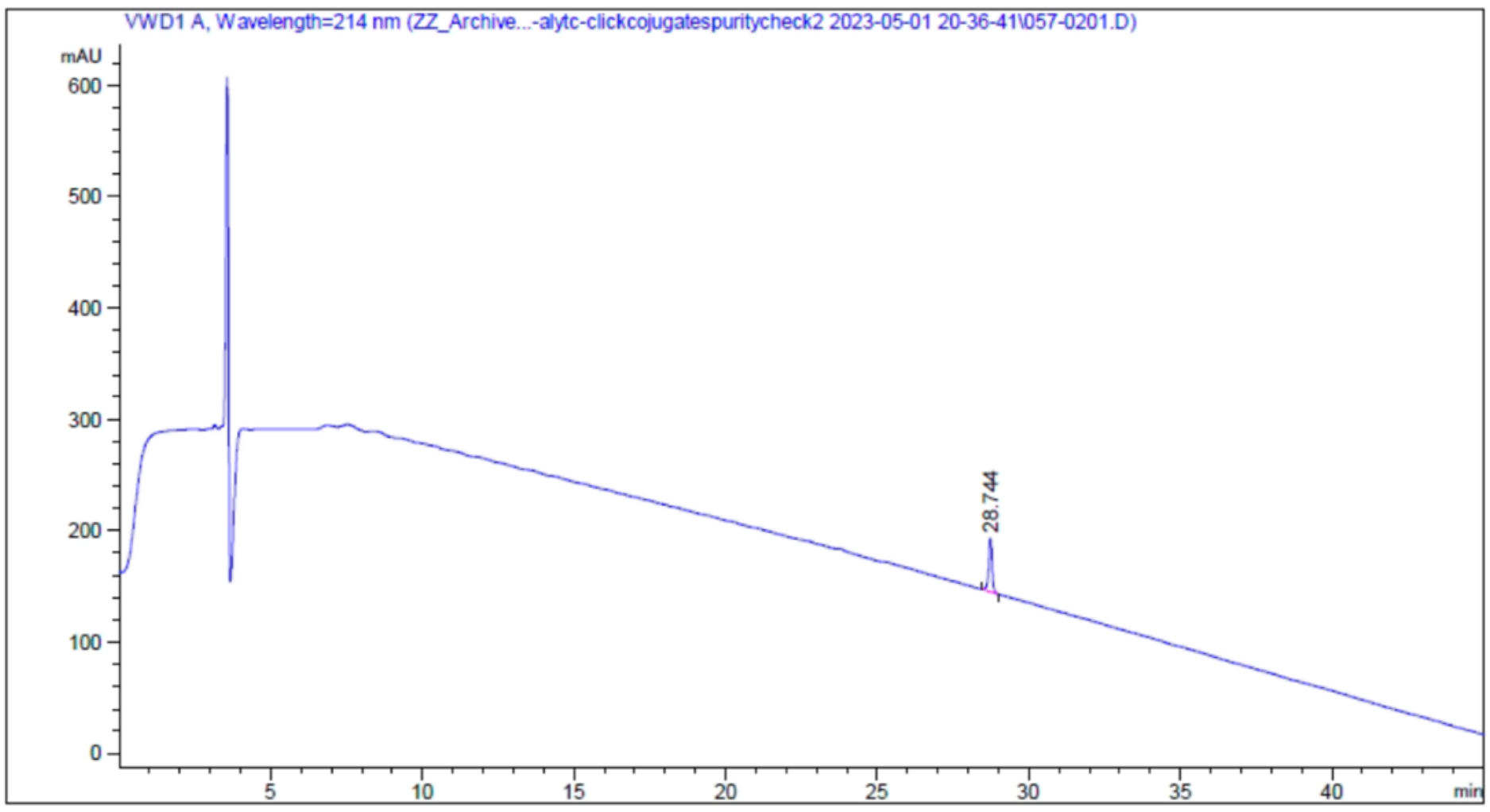

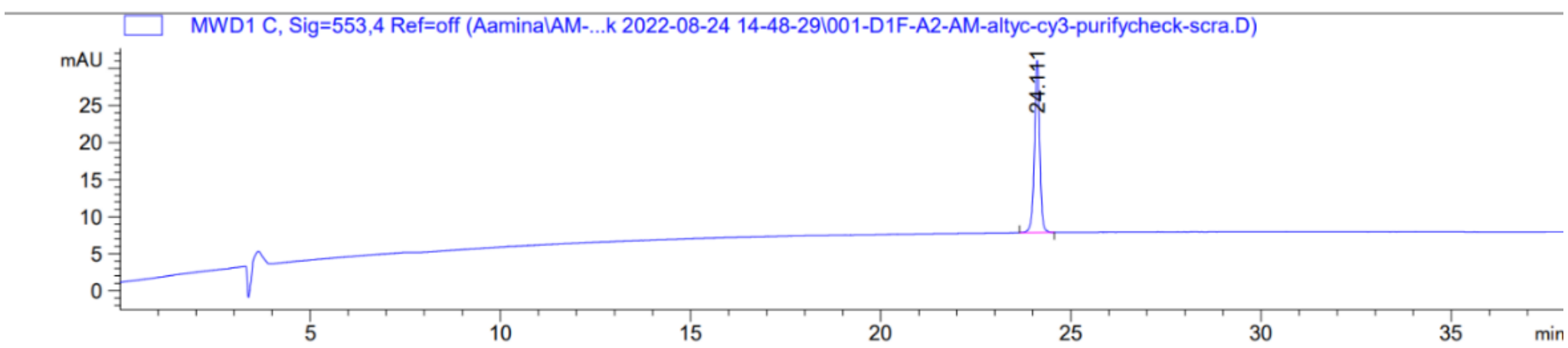

Document S3

CD measurements

Far-UV CD spectra of the peptides were obtained using a Chirascan Plus spectrometer (Applied Photophysics, Leatherhead, UK) with samples maintained at 293 K. The far-UV CD spectra were recorded from 260 to 180 nm, 2nm Spectral Bandwidth, 1nm step-size and a 1.2s instrument time per point. Peptides concentration was kept at 40μM and a 0.5 mm cuvette was used for each of the three solvent conditions: 10mM TrisHCl, pH6, 100% v/v TFE, 10mM TrisHCl, 10mM SDS, pH6. In processing, where appropriate, all spectra were buffer & solvent subtracted and Savitsky–Golay smoothing with a convolution width of 4 points applied for better presentation. Spectra were normalised for concentration, pathlength & presented in terms of Mean Residue Ellipticity. Peptide's secondary structures content was estimated using BestSel webserver for folding prediction on CD spectra [1](Table S4)

Figure S4 Far-UV CD spectra of Alyteserin 1a (Black), Alyteserin 2c (Red) & scr-Alyteserin 2c (Blue) at 40μM in (A) 5 mM Tris HCl, (B) TFE and (C) 10mM SDS.

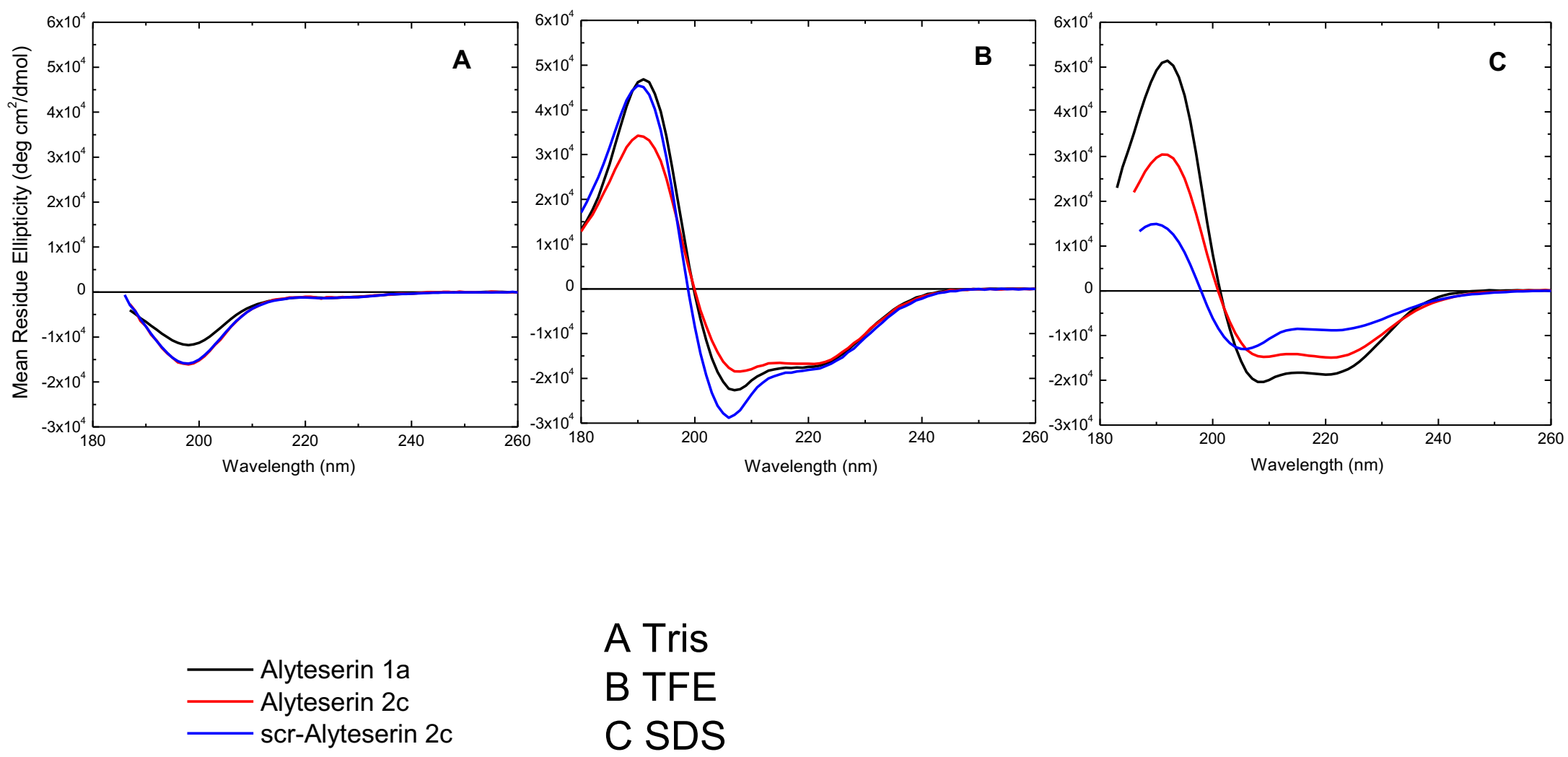

Figure S5 Far-UV CD spectra of peptide as a function of solvents (Black: Tris, Red: TFE, Blue: 10mM SDS): (D) Alyteserin 1a, (E) Alyteserin 2c & (F) scr-Alyteserin 2c at 40μM.

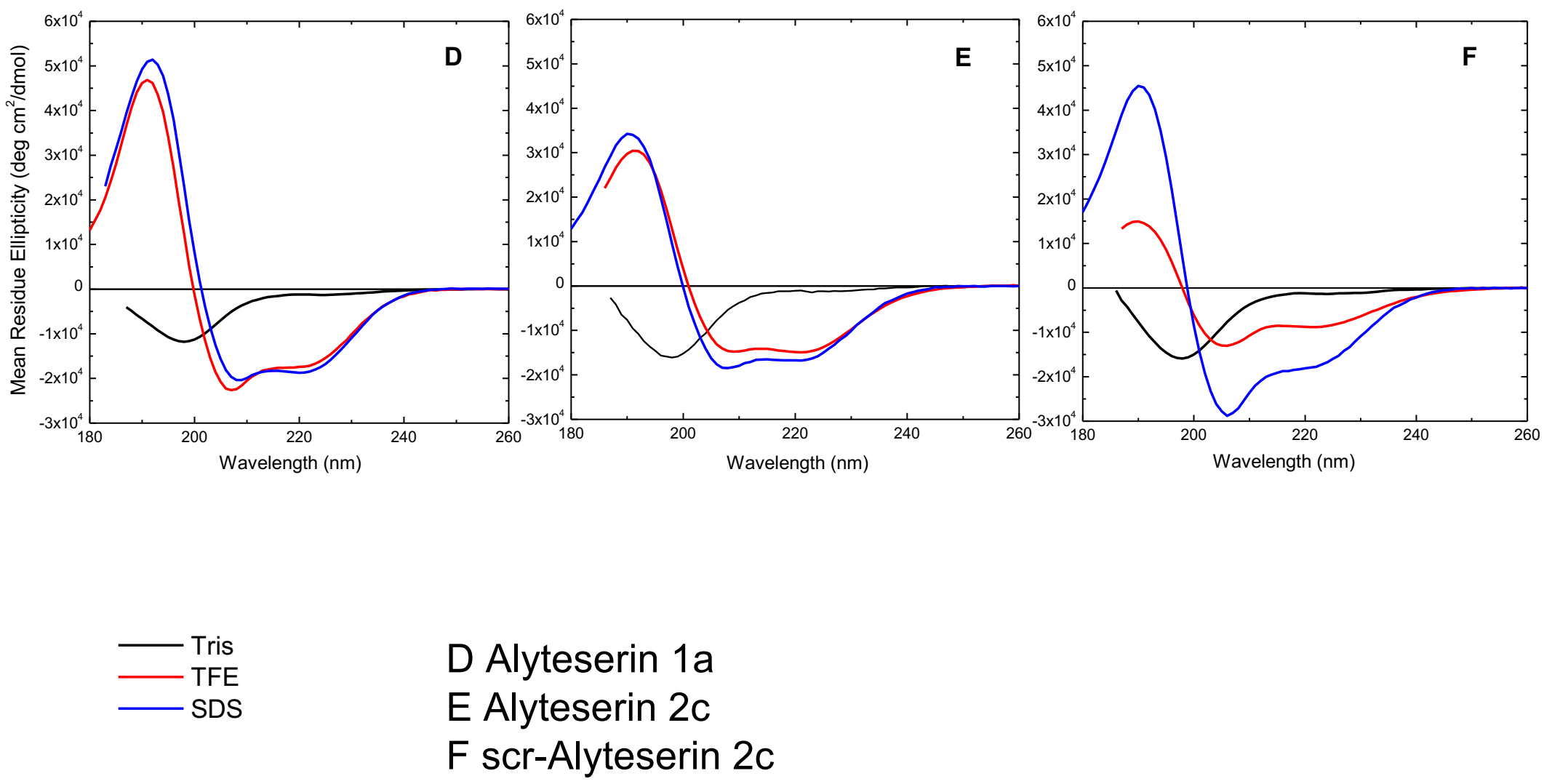

Peptides	% α-Helix		% β–Sheet		% Others	
	TFE	SDS	TFE	SDS	TFE	SDS
Alyteserin 1a	46.7	50.8	12.9	16.7	40.4	32.4
Alyteserin 2c	36.6	26.6	28.9	28.1	36.4	45.3
scr-Alyteserin 2c	42.2	16.6	9.5	29.9	48.3	53.5

Table S4 Conformational properties of the peptides in different solvents

References

1. Micsonai, A., Moussong, E., Wien, F., Boros, E., Vadászi, H., Murvai, N., Lee, Y., Molnár, T., Réfrégiers, M., Goto, Y., Tantos, A., Kardos, J. (2022). BeStSel Webserver for secondary structure and fold prediction for protein CD spectroscopy, *Nucleic Acids Research*, Volume 50(W1), W90–W98
