# Supplementary material for: Early maturation of host antimicrobial peptide defences is associated with host–pathogen coexistence
Source: Nat Chem Biol. 2026 Jul 15;22(11):1731–40. doi: 10.1038/s41589-026-02254-6 (PMC13710165; doi:10.1038/s41589-026-02254-6)
Supplement: Supplementary file 2 [file 41589_2026_2254_MOESM2_ESM.pdf]

nature portfolio

Corresponding author(s): Alethea B Tabor

Last updated by author(s): Mar 20, 2026

Reporting Summary

Nature Portfolio wishes to improve the reproducibility of the work that we publish. This form provides structure for consistency and transparency in reporting. For further information on Nature Portfolio policies, see our Editorial Policies and the Editorial Policy Checklist.

Statistics

For all statistical analyses, confirm that the following items are present in the figure legend, table legend, main text, or Methods section.

n/a	Confirmed	
☐	☒	The exact sample size (n) for each experimental group/condition, given as a discrete number and unit of measurement
☐	☒	A statement on whether measurements were taken from distinct samples or whether the same sample was measured repeatedly
☐	☒	The statistical test(s) used AND whether they are one- or two-sided<br>*Only common tests should be described solely by name; describe more complex techniques in the Methods section.*
☐	☒	A description of all covariates tested
☐	☒	A description of any assumptions or corrections, such as tests of normality and adjustment for multiple comparisons
☐	☒	A full description of the statistical parameters including central tendency (e.g. means) or other basic estimates (e.g. regression coefficient) AND variation (e.g. standard deviation) or associated estimates of uncertainty (e.g. confidence intervals)
☐	☒	For null hypothesis testing, the test statistic (e.g. F, t, r) with confidence intervals, effect sizes, degrees of freedom and P value noted<br>*Give P values as exact values whenever suitable.*
☒	☐	For Bayesian analysis, information on the choice of priors and Markov chain Monte Carlo settings
☒	☐	For hierarchical and complex designs, identification of the appropriate level for tests and full reporting of outcomes
☒	☐	Estimates of effect sizes (e.g. Cohen's d, Pearson's r), indicating how they were calculated

Our web collection on statistics for biologists contains articles on many of the points above.

Software and code

Policy information about availability of computer code

Data collection	None
Data analysis	PEAKS Studio 12, R v 4.2.0, PEPFOLD 3

For manuscripts utilizing custom algorithms or software that are central to the research but not yet described in published literature, software must be made available to editors and reviewers. We strongly encourage code deposition in a community repository (e.g. GitHub). See the Nature Portfolio guidelines for submitting code & software for further information.

Data

Policy information about availability of data

All manuscripts must include a data availability statement. This statement should provide the following information, where applicable:

- Accession codes, unique identifiers, or web links for publicly available datasets
- A description of any restrictions on data availability
- For clinical datasets or third party data, please ensure that the statement adheres to our policy

The mass spectrometry proteomics data have been deposited to the ProteomeXchange Consortium (http://proteomecentral.proteomexchange.org) via the PRIDE partner repository with the dataset identifier PXD069814 (10.6019/PXD069814). All raw RNA reads have been deposited to EBI ENA and NCBI SRA under project accession PRJEB95135, Skin transcriptome assemblies for the post-metamorphic and larval A.obstetricans have been deposited to Zenodo at 10.5281/zenodo.17463202 and 10.5281/zenodo.17463248 respectively. All R scripts and processed peptide data are available at DOI: 10.5061/dryad.k0p2ngfn6.

Research involving human participants, their data, or biological material

Policy information about studies with human participants or human data. See also policy information about sex, gender (identity/presentation), and sexual orientation and race, ethnicity and racism.

Reporting on sex and gender	Not applicable- the research did not involve human participants
Reporting on race, ethnicity, or other socially relevant groupings	Please specify the socially constructed or socially relevant categorization variable(s) used in your manuscript and explain why they were used. Please note that such variables should not be used as proxies for other socially constructed/relevant variables (for example, race or ethnicity should not be used as a proxy for socioeconomic status).<br>Provide clear definitions of the relevant terms used, how they were provided (by the participants/respondents, the researchers, or third parties), and the method(s) used to classify people into the different categories (e.g. self-report, census or administrative data, social media data, etc.)<br>Please provide details about how you controlled for confounding variables in your analyses.
Population characteristics	Describe the covariate-relevant population characteristics of the human research participants (e.g. age, genotypic information, past and current diagnosis and treatment categories). If you filled out the behavioural & social sciences study design questions and have nothing to add here, write "See above."
Recruitment	Describe how participants were recruited. Outline any potential self-selection bias or other biases that may be present and how these are likely to impact results.
Ethics oversight	Identify the organization(s) that approved the study protocol.

Note that full information on the approval of the study protocol must also be provided in the manuscript.

Field-specific reporting

Please select the one below that is the best fit for your research. If you are not sure, read the appropriate sections before making your selection.

☐ Life sciences ☐ Behavioural & social sciences ☒ Ecological, evolutionary & environmental sciences

For a reference copy of the document with all sections, see nature.com/documents/nr-reporting-summary-flat.pdf

Ecological, evolutionary & environmental sciences study design

All studies must disclose on these points even when the disclosure is negative.

Study description	Epizootic outbreaks of invasive pathogens, such as the fungal pathogen Batrachochytnum dendrobatidis (Bd). are increasingly recognised as a global threat to biodiversity. However, not all susceptible host populations are equally Impacted by these disease outbreaks. While some populations persist despite intense infections and high mortality rates. others can transition to enzootic coexistence with the pathogen. Here, we use comparative peptidomics to identify differences in antimicrobial peptide (AMP) maturation in the Bd-susceptible Common midwife toads (Alytes obstetricans) through metamorphosis. Here we show that animals which metamorphose prior to AMP immune maturation display a low diversity AMP phenotype which is deficient on antl·Bd AMPs Where a high incidence of post-metamorphic animals occur with immature AMPs, populations are associated with epizootic disease dynamics. Conversely, populations associated with mature AMPs are stable following invasion of the pathogen. Our results show that even intrinsically susceptible amphibian species can possess the tools which allow populations to recover following a severe Bd epizootic.
Research sample	Skin swabs and skin mucosal samples from Alytes obstetricans
Sampling strategy	Field sampling took place between the 29th July and 6th August 2019, and the 3rd August and 9th August 2020. Animals were captured by hand or net, and we sampled a maximum of 20 tadpoles and 20 post-metamorphic individuals per site (Tab.S3). Recently metamorphosed individuals were found beneath cover objects bordering the lakes and any observed mortalities were recorded. These post metamorphic animals only congregate near water for a few weeks before dispersing into strictly terrestrial habitats, which limits demographic structuring of the animals sampled. Tadpoles had their mouthparts swabbed with a MW10O cotton-tipped swab (Medical Wire and Equipment Co Ltd, UK) and post-metamorphic animals were swabbed according to a standard protocol; each specimen was swabbed by taking five strokes down the centre of the underside, on each flank, the inside of the hind legs and bottom of the feet. Swabs were stored below 4°C until DNA extraction. Peptide sampling was performed using a bath-exposure of norepinephrine. Briefly, each individual was exposed to a 200µM bath of norepinephrine bitartrate (A0397, Merck, UK) and HPLC grade water (V270733, Merck, UK) in a sterile Whirlpak bag (Nasco sampling, US). After 15 minutes, the animal was removed from the Whirlpak and returned to its individual capture bag. The sample was then acidified by addition of 0.2 ml trifluoroacetoc acid (302031, Merck, UK), returned from the field on ice and stored at -18°C until purification for mass spectrometry analysis. Each animal was contained on a new plastic bag and handled with a new pair of powder-free nitrile gloves to prevent cross-infection of individuals.
Data collection	Proteomic analysis of the semi-purified amphibian skin secretions was performed using a Vanquisher LC system connected to a Q· Exactive plus quadrupole-orbitrap mass spectrometer (Thermo Scientific, USA). Lyophilised skin secretions were re-suspended in l00µL solvent (5% acetonitrile in water, 0.1 % formic acid), centrifuged for five minutes at 13,000 rpm. 20 µL of each sample was injected onto a Hypersil Gold, 150 mm x 2.1 mm, 3 µm particle size (P/N 25002-1521300) column. The chromatographic peptides

separation on this column was achieved using Mobile phase A (water, 0.1% formic acid) and Mobtle phase B (acetonitrile, 0.1% formic acid). Twenty minutes gradient was as follows:· After 2 min with 5% B, the proportion or B was raised to 99% B over the next 15 min, and maintained at 99% B for a further 3 min, after within 6 sec the proportion of B was changed to 5% for 1.9 min to equilibrate the Hypersil C18 column. The flow rate was 0.2 ml/min. The column effluent was continuously directed to the HESI source or the Q Exactive mass spectrometer. The HESI probe was operated with a sheath gas flow at 25, an auxiliary gas flow at 5, a spray voltage at 3.80, a capillary temperature at 270 °C, a S-lens RF level at 55, an auxiliary gas heater temperature was set at 50°C The Q Exactive mass spectrometer was operated in a positive ion mode. MS data was acquired using a data-dependent acquisition mode, and operated at 70,000 resolution (full width at half-maximum height, FWHM definition), and the top eight most abundant multiply charged (z ≥2) ions in the 300-1,800 m/z range were selected for MS/MS, with dynamic exclusion enabled 30 sec. The automatic gain control for the Q Exactive was set to 500,000 ions, and the automatic gain control for the MS/MS on the ion trap was set at 8,000 ions. For MS/MS, the Isolation width was set at 1.4 and the collision energy was 35%. MS/MS scans were acquired at a resolution of 17,500 at 200 m/z, the fixed first mass was set to 120 m/z. Three MS/MS microscans for each precursor were accumulated. Dynamic exclusion was enabled, and selected ions were excluded for 20 s before they could be selected for another round of MS/MS.

Timing and spatial scale: 2 week sampling period, one sampling event per year over 2 years across 4 lakes in the Pyrenees

Data exclusions: N/A

Reproducibility: All animals which were characterised were included in the study. Raw data as well as processed data has been provided for the peptidomics

Randomization: All data was from field samples which were not divided into groups.

Blinding: Not applicable

Did the study involve field work? ☒ Yes ☐ No

Field work, collection and transport

Field conditions: Samples taken from four lakes in the Pyrenees

Location: Western Pyrenean Mountain range

Access & import/export: Field sampling in Spain was conducted under permits INAGA (ref. 500201/24/2019/00181) and INAGA (ref. 500201/24/2020/00544) Field sampling in France was conducted under permits issued by Pare National des Pyrenees.

Disturbance: Non-invasive sampling procedures. Access to field sites was via existing hiking trails

Reporting for specific materials, systems and methods

We require information from authors about some types of materials, experimental systems and methods used in many studies. Here, indicate whether each material, system or method listed is relevant to your study. If you are not sure if a list item applies to your research, read the appropriate section before selecting a response.

Materials & experimental systems

n/a	Involved in the study	
☒	☐	Antibodies
☒	☐	Eukaryotic cell lines
☒	☐	Palaeontology and archaeology
☐	☒	Animals and other organisms
☒	☐	Clinical data
☒	☐	Dual use research of concern
☒	☐	Plants

Methods

n/a	Involved in the study	
☒	☐	ChIP-seq
☒	☐	Flow cytometry
☒	☐	MRI-based neuroimaging

Animals and other research organisms

Policy information about studies involving animals; ARRIVE guidelines recommended for reporting animal research, and Sex and Gender in Research

Laboratory animals: 6 Alytes obstetricans were used for developing RNAseq references

Wild animals	We sampled skin secretions from a total of 154 A. obstetricans tadpoles and 123 post-metamorphic individuals across the four survey sites. The amphibians were released unharmed after samphng the skin secretions
Reporting on sex	N/A
Field-collected samples	Skin secretion samples were acidified by addition of 0.2 ml trifluoroacetic acid (302031, Merck, UK), returned from the field on ice and stored at -18°C until purification for mass spectrometry analysis.
Ethics oversight	Ethical approval was given by the ZSL Institute of Zoology ethics committee (512-IoZ23)

Note that full information on the approval of the study protocol must also be provided in the manuscript.

Plants

Seed stocks	N/A
Novel plant genotypes	*Describe the methods by which all novel plant genotypes were produced. This includes those generated by transgenic approaches, gene editing, chemical/radiation-based mutagenesis and hybridization. For transgenic lines, describe the transformation method, the number of independent lines analyzed and the generation upon which experiments were performed. For gene-edited lines, describe the editor used, the endogenous sequence targeted for editing, the targeting guide RNA sequence (if applicable) and how the editor was applied.*
Authentication	*Describe any authentication procedures for each seed stock used or novel genotype generated. Describe any experiments used to assess the effect of a mutation and, where applicable, how potential secondary effects (e.g. second site T-DNA insertions, mosaicism, off-target gene editing) were examined.*
